# Crystal Structure of Glyceraldehyde-3-Phosphate Dehydrogenase from the Gram-Positive Bacterial Pathogen *A. vaginae*, an Immunoevasive Factor that Interacts with the Human C5a Anaphylatoxin

**DOI:** 10.3389/fmicb.2017.00541

**Published:** 2017-04-10

**Authors:** Javier Querol-García, Francisco J. Fernández, Ana V. Marin, Sara Gómez, Daniel Fullà, Cecilia Melchor-Tafur, Virginia Franco-Hidalgo, Sebastián Albertí, Jordi Juanhuix, Santiago Rodríguez de Córdoba, José R. Regueiro, M. Cristina Vega

**Affiliations:** ^1^Integrated Protein Science for Biomedicine & Biotechnology and Ciber de Enfermedades Raras, Center for Biological Research (CIB-CSIC)Madrid, Spain; ^2^Department of Immunology, Complutense University School of MedicineMadrid, Spain; ^3^Hospital 12 de Octubre Health Research InstituteMadrid, Spain; ^4^Abvance Biotech srlMadrid, Spain; ^5^ALBA Synchrotron, Cerdanyola del VallèsCatalonia, Spain; ^6^IUNICS, University of the Balearic IslandsPalma de Mallorca, Spain

**Keywords:** anaphylatoxin, chemoattraction, complement system, C5a, GAPDH (glyceraldehyde-3-phosphate dehydrogenase), gram-positive pathogen, immunoevasion, x-ray crystallography

## Abstract

The Gram-positive anaerobic human pathogenic bacterium *Atopobium vaginae* causes most diagnosed cases of bacterial vaginosis as well as opportunistic infections in immunocompromised patients. In addition to its well-established role in carbohydrate metabolism, D-glyceraldehyde-3-phosphate dehydrogenase (GAPDH) from *Streptococcus pyogenes* and *S. pneumoniae* have been reported to act as extracellular virulence factors during streptococcal infections. Here, we report the crystal structure of GAPDH from *A. vaginae* (*Av*GAPDH) at 2.19 Å resolution. The refined model has a crystallographic *R*_free_ of 22.6%. *Av*GAPDH is a homotetramer wherein each subunit is bound to a nicotinamide adenine dinucleotide (NAD^+^) molecule. The *Av*GAPDH enzyme fulfills essential glycolytic as well as moonlight (non-glycolytic) functions, both of which might be targets of chemotherapeutic intervention. We report that *Av*GAPDH interacts *in vitro* with the human C5a anaphylatoxin and inhibits C5a-specific granulocyte chemotaxis, thereby suggesting the participation of *Av*GAPDH in complement-targeted immunoevasion in a context of infection. The availability of high-quality structures of *Av*GAPDH and other homologous virulence factors from Gram-positive pathogens is critical for drug discovery programs. In this study, sequence and structural differences between *Av*GAPDH and related bacterial and eukaryotic GAPDH enzymes are reported in an effort to understand how to subvert the immunoevasive properties of GAPDH and evaluate the potential of *Av*GAPDH as a druggable target.

## Introduction

*Atopobium vaginae* is a Gram-positive anaerobic bacterium of the *Coriobacteriaceae* family that is held responsible for about 80% of diagnosed cases of bacterial vaginosis (BV) in humans, a condition that can lead to obstetric complications and gynecological disorders (Polatti, 2012; Datcu, 2014; Xiao et al., 2016). Further, complications arising from *A. vaginae* infections might range from predisposing to opportunistic infections by HIV, type II *Herpes simplex* virus, and *Chlamydia trachomatis* infections (Fredricks et al., 2005; Livengood, 2009) to late abortion (Donati et al., 2010), premature rupture of the amniotic membrane (McDonald et al., 2007), chorio-amnionitis (Fahey, 2008), *post-partum* endometritis (Sweet, 1995; Hillier et al., 1996; Pellati et al., 2008), and failure of *in vitro* fertilization and embryo transfer (Fredricks et al., 2005; Livengood, 2009). Since an effective and long-lasting treatment for BV is not available, with relapses occurring in about 30% of cases within a month after treatment completion, new therapeutic approaches are a necessity. Although the molecular basis for *A. vaginae* infection, survival to currently used antibiotic treatments, or the mechanisms behind the acquisition of resistance to metronidazole showed by most strains (Chan et al., 2012; Machado and Cerca, 2015) are not yet fully understood, they are likely to involve many different genes encoding for biofilm formation activities as well as for proteins with virulence and immunoevasive properties.

Interestingly, phylogenetically diverse bacteria have been shown to encode virulence factors that interfere with the proper function of the human complement system, one of the first barriers of defense against bacterial infections from the innate immune system. One such virulence factor is the glycolytic enzyme D-glyceraldehyde-3-phosphate dehydrogenase (GAPDH, E.C. 1.2.1.12). To perform its glycolytic activity GAPDH requires the nicotinamide adenine dinucleotide (NAD^+^) cofactor that remains tightly bound in the active site. The active site of GAPDH contains also two structurally distinct phosphate-binding sites, the P_i_ and P_s_ sites, where inorganic phosphate and the phosphate group from the glyceraldehyde-3-phosphate (G3P) substrate bind, respectively, during reaction (Skarzyński et al., 1987; Yun et al., 2000). In fact, there are two different P_i_ sites depending on the precise location within the active site of the inorganic phosphate moiety, which are termed the “classical” and “new” P_i_ sites (Yun et al., 2000; Mukherjee et al., 2010).

The anaerobic lifestyle of *A. vaginae* implies that a functional glycolytic pathway might be important to sustain infection, therefore making *A. vaginae* (*Av*) GAPDH an interesting target for the design of potent inhibitors. This proposal parallels previous studies on GAPDH from trypanosomatid parasites such as *Leishmania mexicana* (the causative agent of lepromatous leprosy), *Trypanosoma cruzi* (Chagas' disease) and *T. brucei* (African sleeping sickness). These protozoan parasites cause severely impairing diseases with a heavy death toll worldwide, and the lack of wholly effective treatments for these diseases and the problems associated with current drugs (toxicity effects and resistance) have spurred the study of glycosomal trypanosomatid GAPDH. The rationale for targeting trypanosomatid GAPDH for drug discovery relies in the almost exclusive reliance of trypanosomatids on the glycolysis to produce ATP. Indeed, the structures of glycosomal GAPDH from trypanosomatid parasites have been identified as potential drug targets because there are substantial differences in the cavity forming the binding site of the adenine ring of NAD^+^ cofactor between the human and glycosomal GAPDH and in nearby hydrophobic cavities (Suresh et al., 2001). Drugs targeting those hydrophobic cavities could inhibit GAPDH through allosteric effects.

In addition to the known roles of GAPDH in the glycolytic pathway, in the last years there has been an increasing awareness that mammalian and bacterial GAPDH enzymes display many other diverse functions. In mammals, GAPDH participates in membrane fusion, microtubule bundling, nuclear RNA export, DNA replication and repair and apoptosis, and it is in these non-glycolytic roles that mammalian GAPDH has been associated with various diseases including cancer (Zhang et al., 2015), viral pathogenesis (Allonso et al., 2015), Huntington's disease (Hwang et al., 2015; Mikhaylova et al., 2016), Parkinson's disease (Liu et al., 2015) and Alzheimer's disease (El Kadmiri et al., 2014). Bacterial GAPDH enzymes have also shown a wide functional repertoire (Henderson and Martin, 2014). In bacterial pathogens encoding type III secretion systems, GAPDH has been shown to be transported from the bacterial cell to the mammalian host cell through the syringe system (Aguilera et al., 2012), where GAPDH perturbs the redox balance of the attacked host cell leading to its demise or to the creation of more favorable environmental conditions for the invasive bacterial cells. It is significant that GAPDH from many different bacteria is exported to the bacterial cell wall and the extracellular space, where it seems to acquire functions that facilitate infection through direct binding to innate immune proteins. This function has been reported for *S. pyogenes* and *S. pneumoniae* through the interaction of GAPDH with the human C5a anaphylatoxin, which is implicated in inflammatory processes contributing to both the innate and adaptive immune response, and with the C1q complement factor that mediates interactions with IgG and IgM (Terrasse et al., 2012), respectively. In the former case, a solvent-exposed segment of *S. pyogenes* GAPDH spanning the N-terminal 1−165 amino acid residues was identified as the likely docking sequence for C5a (Terao et al., 2006). Other glycolytic enzymes have also been demonstrated to undergo translocation to the extracellular space where they acquire radically different functions, oftentimes pro-infection roles (Agarwal et al., 2012). It is intriguing to consider the possibility that *Av*GAPDH might also be involved in fostering *A. vaginae* infection after its secretion or translocation to the outside of the bacterial cells by diminishing the ability of neutrophils to locate and destroy the pathogen, lowering its antibody-mediated clearance, or perturbing the normal immune response by other unexpected pathways. Providing answers to these open questions will hinge on a more thorough understanding of the structure of *Av*GAPDH and its moonlighting functions in an infection context.

Therapeutic action on the glycolytic or virulence-associated functions of GAPDH represents an attractive route to help curb bacterial infections, while it depends on the availability of specific drugs that can distinguish human GAPDH from the targeted pathogen. The determination of the crystal structure of *Av*GAPDH to 2.19-Å resolution provides a detailed description of the overall structure of this enzyme as well as a high-quality view into the active site. By comparing the *Av*GAPDH structure with those of other pathogens and the available human GAPDH structures, we have observed differences that provide insights into the design of selective inhibitors. Furthermore, the analysis of the peptide segment that corresponds to the C5a-binding motif described for *S. pyogenes* GAPDH hinted at the possibility that GAPDH enzymes from other Gram-positive pathogenic bacteria could participate in the same strategy of infection regardless of the exact colonization environment in the host organism (e.g., throat, lungs, vaginal flora). Binding assays with recombinant C5a and cell migration inhibition assays by surface-immobilized *Av*GAPDH of granulocytes challenged with zymosan-activated serum (where native C5a is produced) have confirmed that *Av*GAPDH could functionally act as a virulence factor, a feature that might contribute to the infectivity and survival of *A. vaginae*.

## Materials and methods

### Cloning

Genomic DNA from an *A. vaginae* type strain isolated from the human vaginal flora of a healthy individual was purchased from the DSMZ (DSM-15829). The gene encoding the 356-amino-acid residue *Av*GAPDH (UniProt Accession No. F1T6A5) was constructed by a two-step PCR protocol designed to incorporate in the PCR amplicon an additional N-terminal hexahistidine tag and a tobacco etch virus cleavage (TEV) site in frame with the *Av*GAPDH gene. This PCR fragment was then inserted into pET21b (Novagen) by restriction-ligation cloning. In addition to the full-length construct, a PCR-amplified DNA sequence encoding only the N-terminal fragment of *Av*GAPDH (residues 1−185) was subcloned in pGEX-6P-1 (GE Healthcare) fused in-frame with the glutathione-S-transferase (GST) gene and a 3C protease cleavage site. Expression plasmids for hexahistidine-tagged *S. pyogenes* (*Sp*) GAPDH (UniProt Accession No. P68777) and *C. perfringens* (*Cp*) GAPDH (UniProt Accession No. Q0STD4) were constructed similarly to that of full-length *Av*GAPDH.

### Protein expression and purification

The expression plasmids were transformed into *E. coli* Rossetta(DE3)pLysS cells (Novagen) and transformants isolated in selective LB-agar plates. Transformants from a freshly prepared plate or from a bacterial glycerol stock were used to inoculate a 40-ml overnight starter culture from which a larger culture (2 l) was initiated the next morning. The culture was allowed to grow at 37°C in LB or Power Broth (Athena) media supplemented with 100 μg/ml ampicillin and 35 μg/ml chloramphenicol to an absorbance of 0.6−0.8 at 600 nm and the temperature was lowered to 20°C for 1 h before induction. The culture was then induced with 0.5 mM isopropyl β-D-thiogalactopyranoside (IPTG) for 18 h. Cells were harvested by centrifugation at 6,000 g for 20 min at 4°C and either used immediately or stored at −80°C until use.

For the hexahistidine-tagged full-length *Av*GAPDH, the cell pellet from 2 l of culture was resuspended in 40 ml of a buffer containing 50 mM Tris−HCl pH 8.0, 500 mM NaCl, 20 mM imidazole and 1 mM phenylmethylsulfonyl fluoride (PMSF), and cells were lysed by sonication. The lysate was centrifuged at 20,000 × g for 30 min at 4°C and clarified further by filtration through a 0.22 μm membrane. The purification procedure consisted of a capture step by affinity chromatography in which the clarified lysate containing N-terminal hexahistidine *Av*GAPDH was loaded on a 5-ml HisTrap column (GE Healthcare) charged with nickel chloride. *Av*GAPDH was eluted using a linear gradient of increasing imidazole concentration (250 mM) over 20 column volumes. Fractions containing *Av*GAPDH were pooled and dialyzed against a buffer containing 50 mM Tris−HCl pH 8.0, 150 mM NaCl. Finally, *Av*GAPDH was subjected to gel filtration chromatography over a HiLoad 16/60 Superdex 200 equilibrated in a buffer consisting in 10 mM Tris−HCl pH 7.5, 150 mM NaCl. We typically obtained 40 mg/l culture of pure *Av*GAPDH with high purity, >95% pure on a Coomassie brilliant blue-stained SDS-PAGE gel (Supplementary Figure 6A). Comparison of the elution volume of *Av*GAPDH with a calibration curve constructed using high and low molecular weight calibration kits (GE Healthcare), following the recommended calibration mixtures following the manufacturer's instructions, corroborated that the quaternary structure of *Av*GAPDH corresponds to a homotetramer (Supplementary Figure 6B). Finally, *Av*GAPDH was dialyzed against a salt-free buffer (10 mM Tris−HCl pH 7.5), concentrated to 30 mg/ml, dispensed in 30-μl aliquots, snap-frozen in liquid nitrogen and stored away at −80°C. Expression (50 ml) and purification of hexahistidine-tagged full-length *Sp*GAPDH and *Cp*GAPDH was carried out as indicated for *Av*GAPDH.

For the GST-3C-fused *Av*GAPDH truncated protein (residues 1−185), the cell pellet from 100 ml of culture was resuspended in 10 ml of a buffer containing 50 mM Tris−HCl pH 8.0, 150 mM NaCl, 10 mM EDTA, and 1 mM PMSF, and cells were lysed by sonication. The lysate was centrifuged at 20,000 × g for 30 min at 4°C and clarified further by filtration through a 0.22 μm membrane. The clarified lysate was then loaded onto a column packed with 3 ml Glutathione Sepharose 4B beads (GE Healthcare), washed thoroughly with binding buffer to baseline absorbance, and on-column tag cleavage was then performed by overnight incubation with 3C protease at 4°C. Purified protein was recovered in the flow-through, concentrated, and applied onto a Superdex 200 10/300GL Increase gel filtration column equilibrated in a buffer consisting in 10 mM HEPES−NaOH pH 7.4, 150 mM NaCl, 3.4 mM EDTA. Peak fractions were analyzed by SDS-PAGE electrophoresis and those fractions corresponding to the estimated molecular mass for monomeric *Av*GAPDH 1−185 were pooled together for further analyses.

The recombinant 74-amino-acid residue human C5a anaphylatoxin (including the C-terminal Arg74) used in this study can be obtained from Crelux GmbH.

### Crystallization

Before attempting crystallization of *Av*GAPDH the required number of aliquots was quickly thawed and centrifuged at 10,000 × g for 10 min at 4°C to remove any potential aggregates that might have resulted from a freezing-thawing cycle. A commercial Pre-Crystallization Test (Hampton Research) was used to adjust the protein concentration to a suitable concentration for more extensive crystallization screenings, which was finally set to 7.5 mg/ml. The full JCSG-plus sparse matrix and the PACT *premier* systematic PEG/Ion/pH screenings (Molecular Dimensions) were performed by the sitting-drop vapor-diffusion method using drops containing 1 μl *Av*GAPDH at 7.5 mg/ml supplemented with 0.6 mM NAD^+^ and 1 μl crystallization condition at 20°C. Moderately sized, plate or cube-shaped crystals of *Av*GAPDH (100−200 μm in their longest dimension) appeared within 1 week under several conditions from both commercial screenings. Two conditions were selected for diffraction studies, condition 1 (0.1 M sodium citrate pH 5.5, 20% PEG 3000) and condition 2 (0.2 M sodium nitrate, 0.1 M Bis-Tris propane pH 8.5, 20% PEG 3350). Crystals were then cryoprotected with 20% (v/v) sterile glycerol, mounted in standard MicroMount (MiTeGen) and flash frozen in liquid nitrogen.

### Data collection and processing

Diffraction data were collected from flash-frozen crystals at 100 *K* using a photon-counting Pilatus 6M (DECTRIS Ltd, Baden, Switzerland) at the BL13-XALOC beamline (ALBA, Barcelona, Spain; Juanhuix et al., 2014). Crystals grown in either condition 1 or 2 diffracted to moderate-to-high resolution (2.19−2.5 Å) and complete data sets were collected from both crystal forms, indexed and processed with XDS (Kabsch, 2014) and scaled and merged with Aimless (Evans and Murshudov, 2013) from the *CCP4* software suite (Winn et al., 2011). Crystals grown under either condition were highly isomorphic, belonging to space group *P*2_1_, with unit-cell dimensions *a* = 71.4, *b* = 132.1, *c* = 85.3 Å, and β = 104.9°. Based on the data merging statistics we selected condition 1 for structure determination. The Matthews coefficient, V_M_, is 2.43 Å^3^/Da, indicates an approximate solvent content of 49.3%. Merging of symmetry-related reflections resulted in 77,410 unique reflections and a multiplicity of 6.7 (6.4 in the last shell, 2.27−2.19 Å) with an *R*_meas_ value of 0.126 (0.904) and CC_1/2_ of 0.997 (0.881). The completeness of the data to 2.19 Å was 98% (89%). Data collection and processing statistics are summarized in Table 1.

**Table 1:** **Crystallographic data collection and refinement statistics**.

	***Av*GAPDH Holoenzyme (PDB 5LD5)**
**DATA COLLECTION**	
Diffraction source	ALBA BL13-XALOC
Wavelength (Å)	0.9795
Temperature (*K*)	100
Space group	*P* 2_1_
**Cell dimensions**	
*a, b, c* (Å)	71.39, 132.11, 85.32
*α, β, γ* (°)	90, 104.88, 90
Resolution range (Å)	47.71−2.19
	(2.27−2.19)
Total reflections	518,896 (44,351)
Unique reflections	77,410 (6903)
Completeness (%)	99 (90)
Multiplicity	6.7 (6.4)
Mean *I*/σ(*I*)	9.46 (2.01)
*R*_merge_^a^	0.116 (0.822)
*R*_meas_^b^	0.126 (0.894)
Wilson *B*-factor (Å^2^)	37.96
CC_1/2_^c^	0.997 (0.883)
CC^*^^d^	0.999 (0.968)
**REFINEMENT AND VALIDATION**	
Reflections used in refinement	77,208 (7000)
Reflections used for R-free	3864 (351)
*R*_work_^e^	0.177 (0.287)
*R*_free_^f^	0.226 (0.324)
**No. of non-H atoms**	
All	10,929
Protein	10,356
Ligands	248
Water	325
No. of protein residues	1362
**R.m.s. deviations**	
Bond lengths (Å)	0.008
Bond angles (°)	1.13
***B*****-factors (Å^2^)**	
All	46.98
Protein	46.99
Ligands	47.11
Water	46.64
Number of TLS groups	14
**Ramachandran plot**	
Favored (%)	97.1
Allowed (%)	2.9
Outliers (%)	0.0
Rotamer outliers (%)	0.62
Clashscore	6.15

*Values for the highest resolution shell are given in parentheses*.

a *R_merge_ = Σ_h_ Σ_i_ |I_i_(h)– < I(h)>|/Σ_h_ Σ_i_ I_i_(h), where h = (hkl), I_i_(h) is the ith measurement and < I(h)> is the weighted mean of all measurements of I(h)*.

b *R_meas_ = Σ_h_ (n/n−1)^1/2^$\Sigma_{i}^{n}$ |I_i_(h)− < I(h)>|/Σ_h_ Σ_i_ I_i_(h), where n is the number of independent observations of I(h)*.

c *CC_1/2_ is the Pearson correlation coefficient calculated between two random half data sets*.

d CC^*^ is the CC of the full data set against the true intensities, estimated from CC^*^ = [2 CC_1/2_/(1 + CC_1/2_)]^1/2^.

e *R_work_ = Σ_h_ |F_O_−F_C_|/Σ_h_ F_O_, where F_O_ and F_C_ are the observed and calculated structure factor amplitudes of reflection h*.

f *R_free_ is as R_work_, but calculated with 5% of randomly chosen reflections omitted from refinement*.

### Structure determination

The structure was determined by the molecular replacement method using the program PHASER (McCoy et al., 2007) from the PHENIX program suite (Adams et al., 2011). Either the full homotetramer (the biological unit) or a dimer of *Staphylococcus aureus* GAPDH devoid of NAD^+^ (*Sa*GAPDH; PDB ID 3LVF; Mukherjee et al., 2010) were used as search models after mutation of the PDB file according to a sequence alignment (63% identity) with *CHAINSAW* (Stein, 2008). Using the *Sa*GAPDH tetramer a single solution was found in the rotation and translation searches with RFZ and TFZ of 30.0 and 15.4, respectively. Rigid-body refinement as implemented in PHASER elicited only minor improvements in correlation coefficient (CC) and *R* factors. In contrast, using a dimer as the search model led to the identification and subsequent use by PHASER of a significant peak in the Patterson map indicating the presence of pseudo translational symmetry. PHASER then switched on the tNCS-enabled MR search that successfully identified two independent solutions that together assembled the biological homotetramer, with RFZ and TFZ scores of 19.2 and 13.2 for the first dimer plus the second, translationally symmetric, dimer. The solutions were improved by rigid-body refinement as implemented in PHASER, with *R*/*R*_free_ factors of 0.39/0.40 for the tetrameric model and 0.40/0.40 for the solution obtained with two dimeric models. At this stage both solutions were indistinguishable (root-mean-square displacement, r.m.s.d., of 0.001 Å over 1,014 superimposed Cα atoms), hence we selected the solution obtained with a full biological unit for further refinement.

### Model building and crystallographic refinement

An omit map calculated from the model phases before (rigid) refinement or model building showed electron density corresponding to four NAD^+^ molecules, one per protomer. The complete homotetramer was then used for rigid-body and maximum likelihood refinement in the resolution range 47.71−2.19 Å within phenix.refine (Afonine et al., 2012) setting aside 5% of the reflections (selected randomly) to create a data set of test reflections for cross-validation of the refinement procedure. Refinement cycles were interspersed with cycles of manual building (first, placing NAD^+^ and then solvent molecules) and validation with *Coot* (Emsley et al., 2010). Torsion-angle non-crystallographic symmetry restraints were applied during the initial refinement but were removed during the final refinement stages. Upon convergence, the refined *Av*GAPDH model had *R*_work_/*R*_free_ = 0.177/0.226 with good stereochemistry. The final model consists of 340 residues of each subunit, four NAD^+^ molecules, 325 water molecules and 12 glycerol molecules (from the cryoprotectant solution). Crystallographic refinement statistics are summarized in Table 1.

### Enzyme activity

The *Av*GAPDH enzyme activity was followed spectrophotometrically by the change in absorbance at 340 nm due to NADH formation, according to a previously described method (Ferdinand, 1964). Assays were performed in an Eppendorf BioSpectrometer spectrophotometer at 25°C. A standard assay was carried out in a final volume of 0.15 ml in the presence of 40 mM Tris-HCl pH 8.5 and 2 mM ethylenediaminetetraacetic acid (EDTA), 0.1 μM of purified enzyme, and indicated concentrations of substrates: nicotinamide adenine dinucleotide (NAD^+^), glyceraldehyde-3-phosphate (G3P) and inorganic phosphate (P_*i*_). NAD^+^ concentration was varied between 0.03−2 mM, G3P concentration between 0.6−15 mM, and P_*i*_ concentration between 0.65−24 mM. Michaelis–Menten parameters, *K*_M_ and *V*_max_ were obtained by nonlinear regression fitting of the kinetic data using SigmaPlot v13.0 (Systat Software Inc.).

### Sequence conservation analysis

GAPDH sequences of known structure and with a significant degree of sequence similarity to *Av*GAPDH were found with BLAST (Camacho et al., 2009) using sequences stored in the PDB, aligned with MUSCLE and used to reconstruct a maximum likelihood phylogenetic tree with PhyML (Guindon et al., 2010).

### Ligand blotting

A ligand blotting assay was set up to detect the interaction between *Av*GAPDH and C5a essentially as described before (Terao et al., 2006). Briefly, C5a was biotinylated using the ECL Protein Biotinylation System (Amersham Biosciences) following the manufacturer's instructions. Then, different concentrations of purified *Av*GAPDH were resolved on a 15% SDS-PAGE gel and transferred to nitrocellulose membranes. The membranes were blocked overnight at 4°C with 10% membrane blocking agent and treated with 50 μg/ml biotinylated C5a for 1 h. After extensively washing the membranes and incubating with horseradish peroxidase (HRP)-labeled streptavidin at room temperature for 1 h, C5a-containing bands were revealed with luminol and hydrogen peroxide and subsequently visualized by chemiluminescence on a Luminescent Image Analyzer LAS-3000 at room temperature.

### Cross-linking

Specific cross-linking assays with *Av*GAPDH were performed with bis(maleimido)ethane (BMOE; Pierce 22322), a 7-atom, 8.0-Å short spacer arm cross-linking reagent that generates non-cleavable cross-links between sulfhydryl groups that are in close proximity. Cross-linking reactions were performed in assay buffer (10 mM HEPES−NaOH pH 7.4, 150 mM NaCl, 3.4 mM EDTA) containing 0.3 mM BMOE, incubated overnight at 4°C, and visualized by SDS-PAGE electrophoresis and Coomassie-brilliant blue (CBB) staining.

### Enzyme-linked immunosorbent assays

The C5a interaction with *Av*GAPDH was studied also with an enzyme-linked immunosorbent assay (ELISA). The wells of a polysterene microtiter plate (F96 Maxisorp, Nunc) were coated with 100 μl of 10 μg/ml of purified *Av*GAPDH in 50 mM Tris−HCl pH 8.5 at 4°C overnight. Wells were emptied and washed with TBS-T (Tris-buffered saline pH 7.4 with 0.05% w/v Tween-20) and the free sites on the plastic surface were blocked with 1% BSA (bovine serum albumin) in TBS-T for 2 h at 37°C. Uncoated wells and wells coated with *Av*GAPDH were also blocked. After washing extensively with TBS-T, various dilutions of C5a in TBS (1–100 μg/ml) were added to the *Av*GAPDH-coated wells. The plate was incubated for 3 h at 37°C and then washed 4 times with TBS-T. A mouse anti-human C5a antibody was added at a 1:2,500 dilution in TBS-T (100 μl/well) and the plate was incubated for 1 h at 37°C and washed as before. Next, a goat anti-mouse IgG-HRP conjugate (1:1000 dilution) was added and the plate was incubated at 37°C for 1 h. The unbound conjugate was discarded and the plate was washed 5 times with TBS-T. OPD (*o*-phenylenediamine dihydrochloride) substrate tables were dissolved in double distilled water and added immediately (100 μl/well) to the plate. The absorbance was measured at 495 nm in a microplate reader.

### Immobilization of *Av*GAPDH

Purified full-length of *Av*GAPDH, *Sp*GAPDH, and *Cp*GAPDH, and the 185-amino-acid N-terminal fragment of *Av*GAPDH were immobilized by multipoint covalent binding on to highly functionalized aminoethyl agarose (AM-4) beads (ABT, 40−60 μmol aminoethyl/ml gel, 4% agarose), thereby allowing protein immobilization through coupling with carboxylate groups from exposed aspartate and glutamate residues and the C terminus. The amount of covalently crosslinked GAPDH was calculated from equation 1 below, where *C*_0_ is the protein concentration at starting immobilization time and *C*_*F*_ is the protein concentration at final immobilization time:

(1) $$\begin{array}{r} \text{Immobilization Yield }\left(\text{IY\%}\right)=100\times \;\left(C_{0}\;-\;C_{F}\right)/C_{0} \end{array}$$

According to manufacturer instructions, 50 μl of AM-4 resin was rinsed with water and equilibrated with a buffer consisting of 10 mM Tris-HCl pH 7.6. The equilibrated resin was then activated with 60 mM *N*-(3-dimethylaminopropyl)-*N*'-ethylcarbodiimide (EDC) and next various amounts of protein were incubated with the treated resin at room temperature on an orbital shaker with gentle stirring. At different times aliquots were removed to monitor the amount of unreacted protein until it reached a plateau (which occurred at about 3 h), indicating the saturation of the accessible functional carboxylate groups on the resin. Next, the suspension was centrifuged (13,500 rpm for 2 min) and the pelleted resin was carefully washed with water, 1 M NaCl, and water again.

### Migration assays

After obtaining written informed consent from healthy donors, human granulocytes were isolated from EDTA venous blood samples by centrifugation on a Histopaque-1070/1119 gradient (Sigma-Aldrich). These studies were performed according to the principles expressed in the Declaration of Helsinki and approved by the relevant Institutional Research Ethics Committee. Residual erythrocytes were depleted by incubation with 0.5 ml Ammonium-Chloride-Potassium Lysing Buffer (150 mM NH_4_Cl, 10 mM KHCO_3_, 0.1 mM Na_2_EDTA, pH 7.2) for 2.5 min at 25°C. Cells were washed twice with PBS (400 × g for 6 min at 25°C) and used at 2 × 10^5^ cell/ml in RPMI-1640 (Sigma-Aldrich). Chemotactic migration was evaluated in 24-well transwell plates with 5 μm pore size polycarbonate filters (Corning). 0.2 × 10^6^ cells were loaded in the upper chambers, while lower chambers contained 1 nM C5a (diluted from 48 nM C5a from zymosan-activated human serum, measured by ELISA) pre-incubated for 1 h at RT with 15 μl of either the indicated GAPDH-linked or untreated resin beads in RPMI-1640. Plates were then incubated for 30 min at 37°C with 5% CO_2_. Using forward and side scatter gating, the number of granulocytes recovered from lower chambers was quantified by flow cytometry for 1.5 min (constant flux acquisition, FACSCalibur, BD Biosciences). Normal migration to 1 nM C5a preincubated with untreated resin beads was 5,841 ± 2,141 cells, background migration to EDTA-containing human serum with undetectable C5a by ELISA was 510 ± 245 cells. Migration after preincubation with GAPDH resin beads was expressed relative to normal migration after preincubation with the same amount of untreated resin beads. Statistical analysis and graphical representation were done using the R software.

## Results

### Overall structure

**Figure 1:**
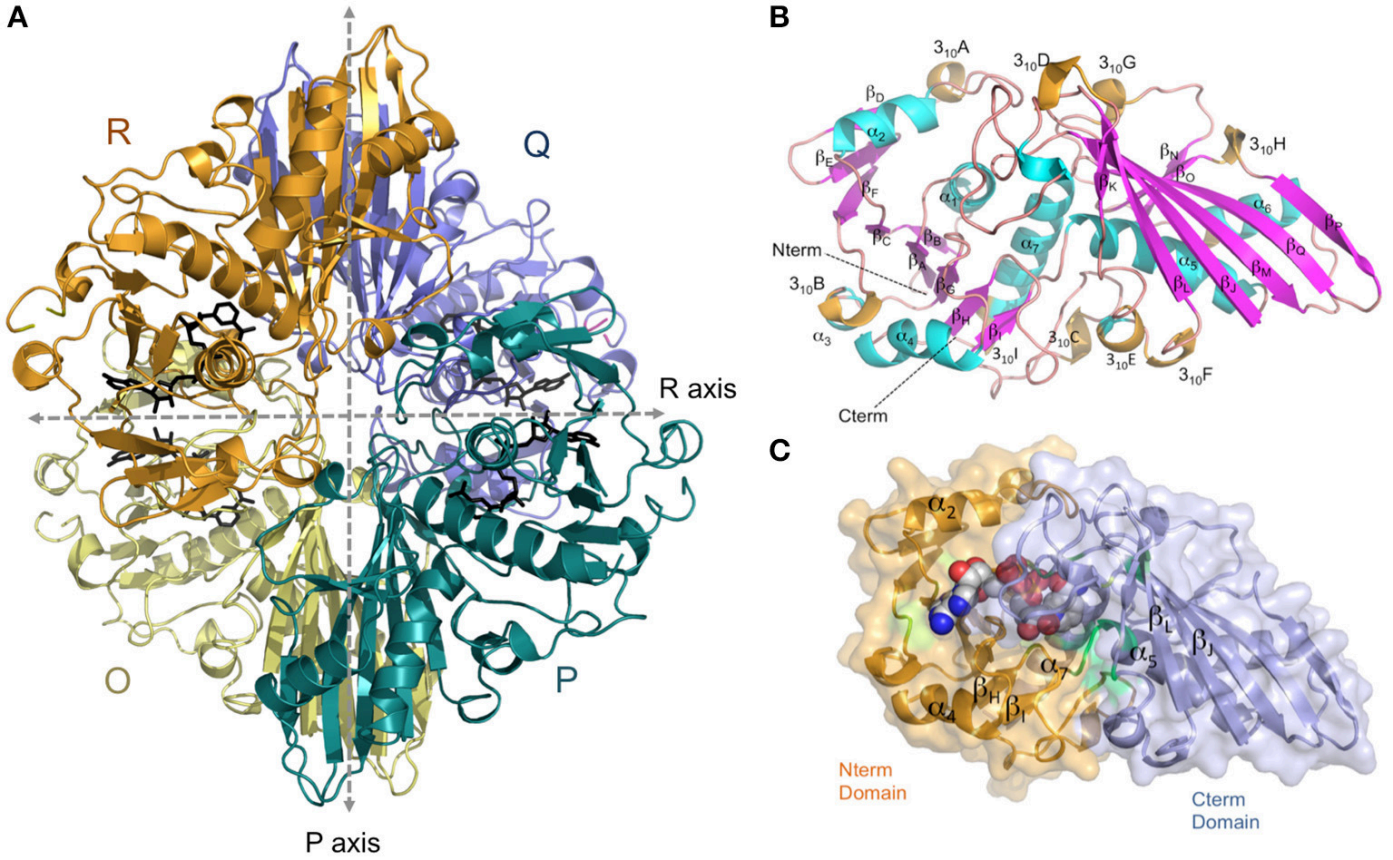
**Overall architecture of the homotetrameric ***Av***GAPDH. (A)** The *Av*GAPDH homotetramer subunits *O, P, Q*, and *R* are shown in yellow, cyan, violet, and orange, respectively. In the active site of each protomer there is one NAD^+^ bound (black). Symmetry axes are shown as dashed arrows, with the *Q* axis shown perpendicular to the plane of the paper. **(B)** Ribbon representation of the *Av*GAPDH monomer with secondary structural elements labeled. Helices are colored in cyan, 3_10_ helices in orange, strands in violet, and interconnecting segments in pink. The cofactor molecule is not shown for clarity. **(C)** Shown in the same orientation as in **(B)**, the two domains of *Av*GAPDH are mapped in two distinct colors on a molecular surface representation. The N-terminal cofactor-binding domain is mapped in orange and the C-terminal catalytic domain in violet. NAD^+^ atoms are depicted as spheres in CPK colors. The figure was produced with PyMOL (Schrodinger LLC).

The crystal structure of *Av*GAPDH has been solved at 2.19 Å resolution and a complete macromolecular model has been built and refined with correct stereochemical parameters. Upon convergence, the crystallographic *R* factors were *R*_work_/*R*_free_ = 0.177/0.226. Table 1 contains the X-ray diffraction data collection and refinement statistics. The overall structure and domain architecture of *Av*GAPDH closely resemble those described in previous GAPDH structures. The quaternary structure shows four subunits designated *O, P, Q*, and *R* with three non-equivalent intersubunit interfaces related by three mutually perpendicular dyad axes (*P, Q*, and *R*; nomenclature consistent with PDB code 1CER; Tanner et al., 1996) and one NAD^+^ molecule bound per active site, which is clearly defined in electron density (Buehner et al., 1974; Roitel et al., 2003). The four monomers in the GAPDH homotetramer are not necessarily equivalent since previous studies have indicated the presence of negative allosteric effects across subunits (Nagradova, 2001). In the final model of *Av*GAPDH, the tetramer shows minor deviations of <0.2° from the ideal 222 (D2) molecular symmetry (Figure 1A). The *Av*GAPDH homotetramer contains three asymmetric interfaces between each protomer and the adjacent subunits. The *O*-*P* interface contains the largest buried solvent area (1,830 Å^2^) and includes about 30 hydrogen bonds and 12 salt bridges. The second largest interface is the *O*-*R* interface, with 1,325 Å^2^, and is formed by 10 hydrogen bonds and 2 salt bridges. Finally, the smallest of the three interfaces, between the *O*-*Q* subunits, covers about 500 Å^2^ and includes 11 hydrogen bonds and 4 salt bridges.

*Av*GAPDH is characterized by an N-terminal NAD^+^-binding domain (residues 1−167) and a C-terminal catalytic domain (residues 168−356; Harris and Waters, 1976). The N-terminal NAD^+^-binding domain features the classical α/β dinucleotide-binding fold or Rossmann fold (Figures 1B,C). It folds into eight β-sheets (β_A_-β_H_) connected by helices and short loops. The helices of the N-terminal domain are α1-α2, which are located before and after β_B_, and three 3_10_ helices: 3_10_A, which immediately precedes β_C_, β_D_, and β_E_, and 3_10_B followed by α3-α4. The third 3_10_ helix (3_10_C) connects an extended section with a central isolated β-bridge centered at Ile147 with the last β-strand (β_H_) of the NAD^+^-binding domain. Overall, the structural tandem β-α-β-α-β motif is conserved (Lesk, 1995; Antonyuk et al., 2003). The C-terminal domain consists of a seven-stranded mixed parallel β-sheet (β_I_-β_P_), three α-helices (α4-α6) there are six 3_10_ helices that are found immediately before or following β-strands (Figures 1B,C). Helices 3_10_D-F are located between β_*J*_-β_K_ strands, while 3_10_G, 3_10_H and 3_10_I are placed after β_J_, β_K_,and β_P_-α7, respectively, with α6 between the β_L_-β_M_ strands. The N-terminal NAD^+^-binding domain and the C-terminal catalytic domain are bridged at the joint between strand β_H_ and the long helix α4. The catalytically active nucleophile Cys168 and the activating His195 lie at the beginning of helix α5 and the end of the following strand, β_I_, respectively. The C-terminal α6 helix is inserted into an extensive groove of the N-terminal domain where it forms a significant part of the interdomain interface and contributes to the binding pocket for the nicotinamide ring of the NAD^+^ cofactor. No traces of a phosphate ion were found bound into the active site.

The distance between the active-site Cys168 sulfhydryl to the Nε2 atom of the active-site His195 is 3.39–3.45 Å in chains *O* and *R* and 3.64 Å in chains *Q* and *R*, which is consistent with the defined closed conformation for the reported structure of homotetrameric *Av*GAPDH. Differences in cofactor binding between the subunits of GAPDH from various sources have been previously postulated as the basis for the negative cooperativity observed in NAD^+^ binding, where the first two NAD^+^ molecules bind with a higher affinity than the third and fourth NAD^+^ molecules (100−300-fold less tightly; Nagradova, 2001), although this asymmetry has not been demonstrated for every GAPDH. In particular, no negative co-operativity was observed in trypanosomatid GAPDH enzymes (Lambeir et al., 1991).

The crystal structure of *Av*GAPDH presents the closed conformation of the enzyme. In support of a closed conformation, the conformation of the NAD^+^-bound *Av*GAPDH is significantly more similar to the structure of NAD^+^-bound *Bacillus stearothermophilus* (*Bs*) GAPDH (PDB ID 1GD1) than to the NAD^+^-free form (PDB ID 2GD1; Skarzyński and Wonacott, 1988) as shown by the corresponding r.m.s.d. values (1.35 Å vs. 1.54 Å, respectively). *Av*GADPH in solution is likely to be more flexible or have more disordered surface loops than the closed enzyme conformation.

In many GAPDH structures a conserved Val residue adopts a highly strained backbone conformation (Kim et al., 1995), within the most disallowed region of the Ramachandran plot, in order to help maintain a hydrogen bond interaction between a critical Asn and the nicotinamide ring of NAD^+^ (Souza et al., 1998). In contrast, the equivalent residue in *Av*GAPDH, Gly257, establishes a hydrogen bond with Asn336 through its main-chain amide that has a similar length (2.9−3.1 Å) and orientation as the more commonly found Val-Asn hydrogen bond. This isofunctional Val to Gly substitution has also been observed in *S. aureus* and *Thermotoga maritima* GAPDH (Korndörfer et al., 1995; Mukherjee et al., 2010).

#### The active site and cofactor-binding site are located in a large conserved groove

An omit map calculated with molecular replacement phases (prior to refinement) around the catalytic Cys168 of all subunits (Supplementary Figure 1) confirms the absence of covalent oxidation modifications that would inactivate *Av*GAPDH, as those present in previously reported GAPDH structures, which have been explained as oxidation of the cysteine residue to sulfonic acid or *S*-oxyl cysteine (Cowan-Jacob et al., 2003). His195, which assists Cys168 by increasing its nucleophilicity, is itself polarized by interactions with the catalytically important residue Arg251 (Nagradova, 2001). The conformation adopted by Arg251 side chain allows it to form hydrogen bonds with Asp200 and the nearby Gln201 side chains, on the opposite side to His195. Similar polarizing interactions between Arg residues with Asp carboxylates have been described to facilitate catalysis by lowering the p*K*_a_ of Arg251 for the guanidinium side chain from 12.5 to 9.0 (Kuzminskaya et al., 1991; Cowan-Jacob et al., 2003).

**Figure 2:**
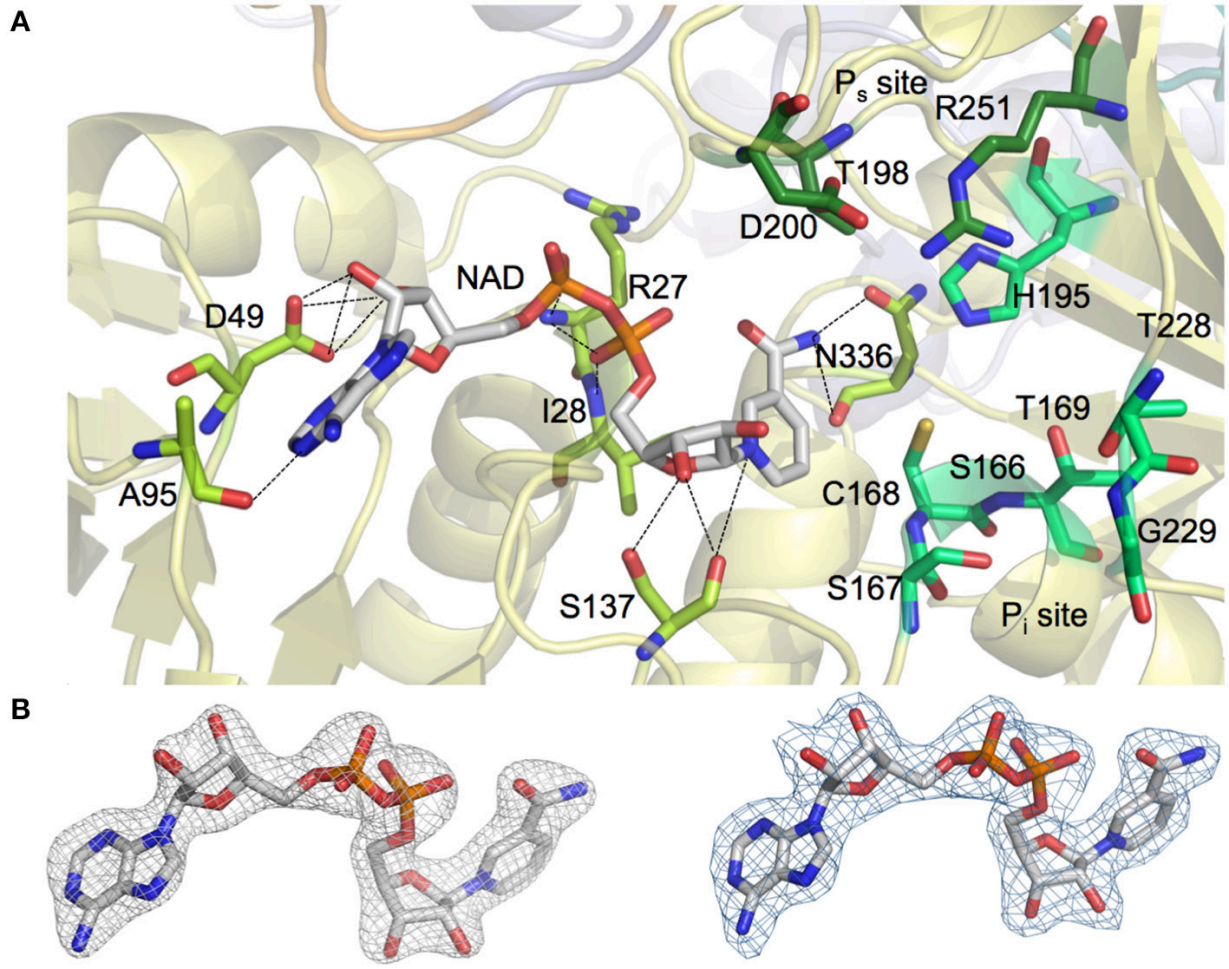
**NAD^**+**^ and phosphate binding sites in ***Av***GAPDH. (A)** Binding pockets for the NAD^+^ cofactor (shown in sticks with carbon atoms in gray and all other atoms in CPK colors) and for the inorganic and substrate phosphate sites (“new P_i_” and P_s_ sites, labeled). The placement of the P_i_ site in *Av*GAPDH as a “new P_i_” site is consistent with the *S. aureus* GAPDH structure (PDB ID 3K73). Enzyme residues in direct contact with NAD^+^ are represented in sticks (carbon atoms in light green) and polar interactions (hydrogen bonds) are depicted as dotted lines. Residues predicted to participate in the stabilization of the inorganic and substrate phosphate groups (including the catalytic Cys168) are also shown as sticks (with carbon atoms in two different shades of dark green). The *Av*GAPDH structural scaffold is shown in ribbons in depth-cued light colors. **(B)** Omit map showing unbiased electron density map of NAD^+^ cofactor in *Av*GAPDH. Unbiased omit map calculated with molecular replacement phases (prior to building the cofactor and solvent structures and prior to refinement) contoured at 1.5 σ revealing the presence of the NAD^+^ cofactor at full occupancy. For comparison, electron density map for the final model of the NAD^+^ cofactor displayed at the same contour level (right). The figure was produced with PyMOL (Schrodinger LLC).

**Figure 3:**
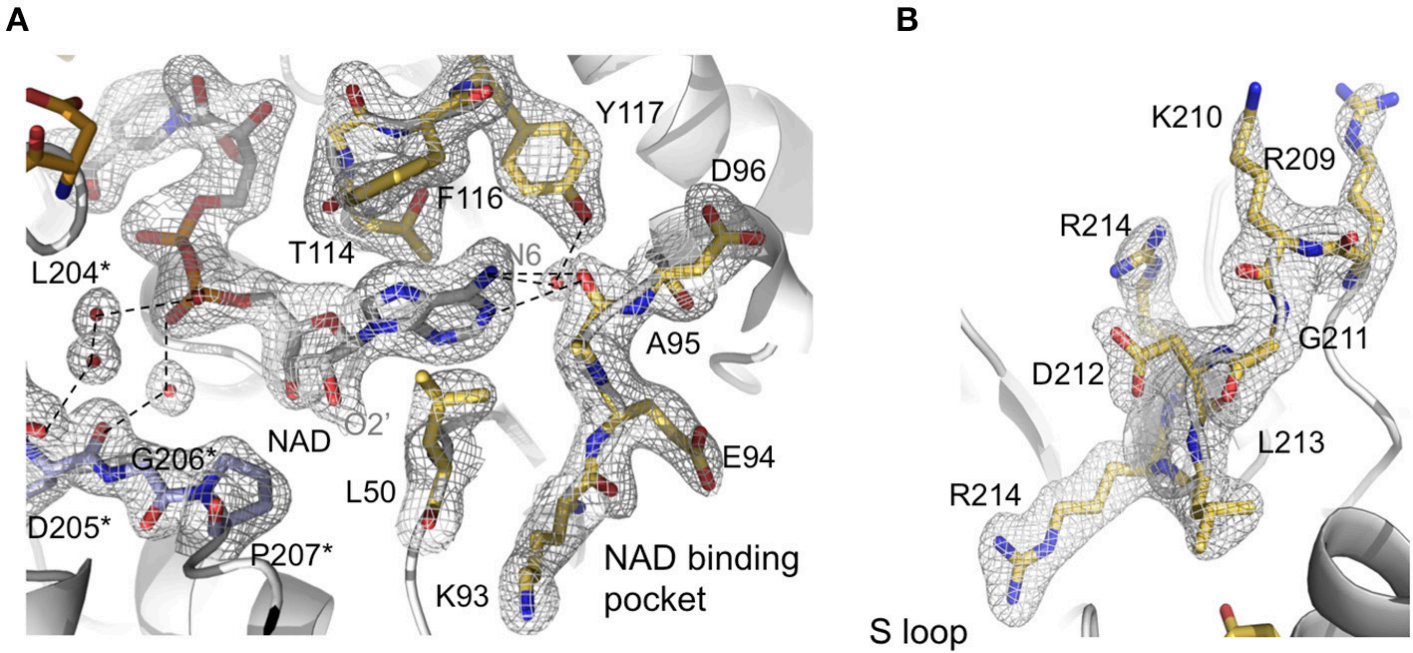
**Active site features of ***Av***GAPDH**. *Av*GAPDH is shown in cartoon representation (gray; NAD^+^ binding site is in gold; and *Av*GAPDH-specific residues in the *S* loop in violet). **(A)** Network of interactions that stabilizes NAD^+^ binding. The main and side chains of NAD^+^-binding residues are shown in stick representation. Key water molecules are shown as red spheres. They are overlaid with the σ_A_-weighted (2*m*F_O_−*D*F_C_) electron density map contoured at 1 σ level. Asterisks denote residues belonging to the neighboring subunit. **(B)** Continuation of the *S* loop (residues Ala196-Ile223, in gold) beyond the NAD^+^ binding site, depicting its elongated, irregular shape. Residues are overlaid with the σ_A_-weighted (2*m*F_O_−*D*F_C_) electron density map contoured at 1 σ level. The figure was produced with PyMOL (Schrodinger LLC).

NAD^+^ molecules are tightly bound in an elongated cleft in all four protomers of the homotetrameric *Av*GAPDH structure (Figures 1A, 2A). The presence and occupancy of NAD^+^ was assessed with unbiased omit maps (Figure 2B). The nicotinamide group is deeply embedded in the protein, as suggested by the similar or even lower *B*-factors of the NAD^+^ molecules (36.8−49.2 Å^2^) in comparison with the average *B*-factors for the protein chain (43.0−52.1 Å^2^). The atomic *B*-factors for the adenine moiety are between 2.0 Å^2^ lower and 8.5 Å^2^ higher than the atomic *B*-factors for the nicotinamide part of the same NAD^+^ molecule, thereby indicating a well-defined conformation for the adenine group in all chains (Figures 2, 3). Furthermore, the electron-density real-space correlation coefficient (RSCC) for the NAD^+^ molecules is 0.94−0.97 (0.90 overall), indicating that the NAD^+^-binding pocket is fully and equally occupied in all four protomers (Figures 2B, 3A). The relatively low *B*-factors and near-unity RSCC for the NAD^+^ molecules argue for a very tight binding of the cofactor into the NAD^+^-binding pocket.

Most residues involved in cofactor binding are completely conserved between *Av*GAPDH and human GAPDH. However, there are several substitutions in the cofactor-binding site, especially in residues located around the entry gateway. Specifically, Pro36, Phe37, and Arg80 in human liver GAPDH are substituted in *Av*GAPDH by Asp49, Leu50, and Ala95, respectively (Figures 2A, 3A). Structurally, the smaller aliphatic side chain of Leu50 fulfills essentially the same role as Phe37 in the human enzyme, although they build slightly different hydrophobic environments that might be exploited by selective drug targeting. The loss of the positively charged Arg80 present in human GAPDH is likely inconsequential for NAD^+^ binding, since Arg80, exactly as Ala95 in *Av*GAPDH, interacts with the cofactor via a main-chain carbonyl hydrogen bond. Interestingly, in *Plasmodium falciparum* (Satchell et al., 2005) the equivalent position is occupied by a Lys, thereby suggesting that a positively charged side chain might play a secondary functional role in NAD^+^ and/or substrate binding/release. A network of water molecules further contributes to stabilizing the bound NAD^+^ conformation in the active site by holding in place the adenosyl and nicotinamide rings and the nucleotide linking phosphate groups (Supplementary Figure 2).

#### Phosphate sites

Since the P_i_ and P_s_ sites (Supplementary Figure 3) supply essential functions, the GAPDH residues involved in shaping them are highly conserved across very divergent species (Supplementary Figure 4). While the P_s_ site has been found at an identical location across all known prokaryotic and eukaryotic GAPDH structures, two distinct P_i_ sites have been described. The so-called “classical P_i_” site is modeled after the *Bs*GAPDH co-crystallized with sulfate anions (PDB ID 1GD1, sequence identity: 50.3%; Skarzyński et al., 1987), whereas a “new P_i_” site was first characterized in *T. maritima* (*Tm*) GAPDH (Korndörfer et al., 1995) and subsequently corroborated in *Escherichia coli* (*Ec*) GAPDH and *S. aureus* (*Sa*) GAPDH structures (Yun et al., 2000; Mukherjee et al., 2010). Their different locations within the GAPDH active site is explained by the differential placement of the β-strand-loop-α-helix stretch spanning residues 226−231, which is more deeply buried in those structures with a “new P_i_” site. The strand-loop-helix motif residues in *Av*GAPDH were all clearly defined in electron density maps. In the *Av*GAPDH structure there is no residual electron density at the P_i_, “new P_i_,” or P_s_ sites, indicating that they were completely unoccupied in the crystal (Supplementary Figure 4B). By analogy with most GAPDHs of known structure that have been crystallized with inorganic phosphate or sulfate, we could assign the four residues that contribute to the canonical P_*s*_ site: Thr195 (Oγ atom), Asp200 (side-chain carboxylate), Arg251 (guanidinium group), and NAD^+^ (2′ hydroxyl of the nicotinamide-ribose part, O2D; Supplementary Figure 3A). A conformational switch involving Arg251 has been proposed to play an important role in the regulation of the substrate G3P binding and the release of the product, 1,3-bisphosphoglycerate (1,3-BPG; Souza et al., 1998). Inspection of the conformation adopted by the 226−231 loop of *Av*GAPDH indicates that its P_i_ site is most likely a “new P_i_” site analogous to that of *Sa*GAPDH, which would recruit the side chains of Ser167, His195, Thr228, and Arg251, and the main-chain carbonyl and side-chain hydroxyl of Thr169 (Supplementary Figures 3B, 4). The neighboring residue Arg215 can interact with the side chain of Asp200 to stabilize its position and, conceivably, could also support a product-release mechanism whereby Arg215 would interact with the phosphate moieties of 1,3-BPG thereby displacing the similar interaction with Arg251, which is part of both the “new P_i_” and the P_s_ sites. This displacement would require the side chains of Asp200 and Arg215 to flip in and out according to the substrate binding state of *Av*GAPDH (Supplementary Figure 4).

#### *S* loop

**Figure 4:**
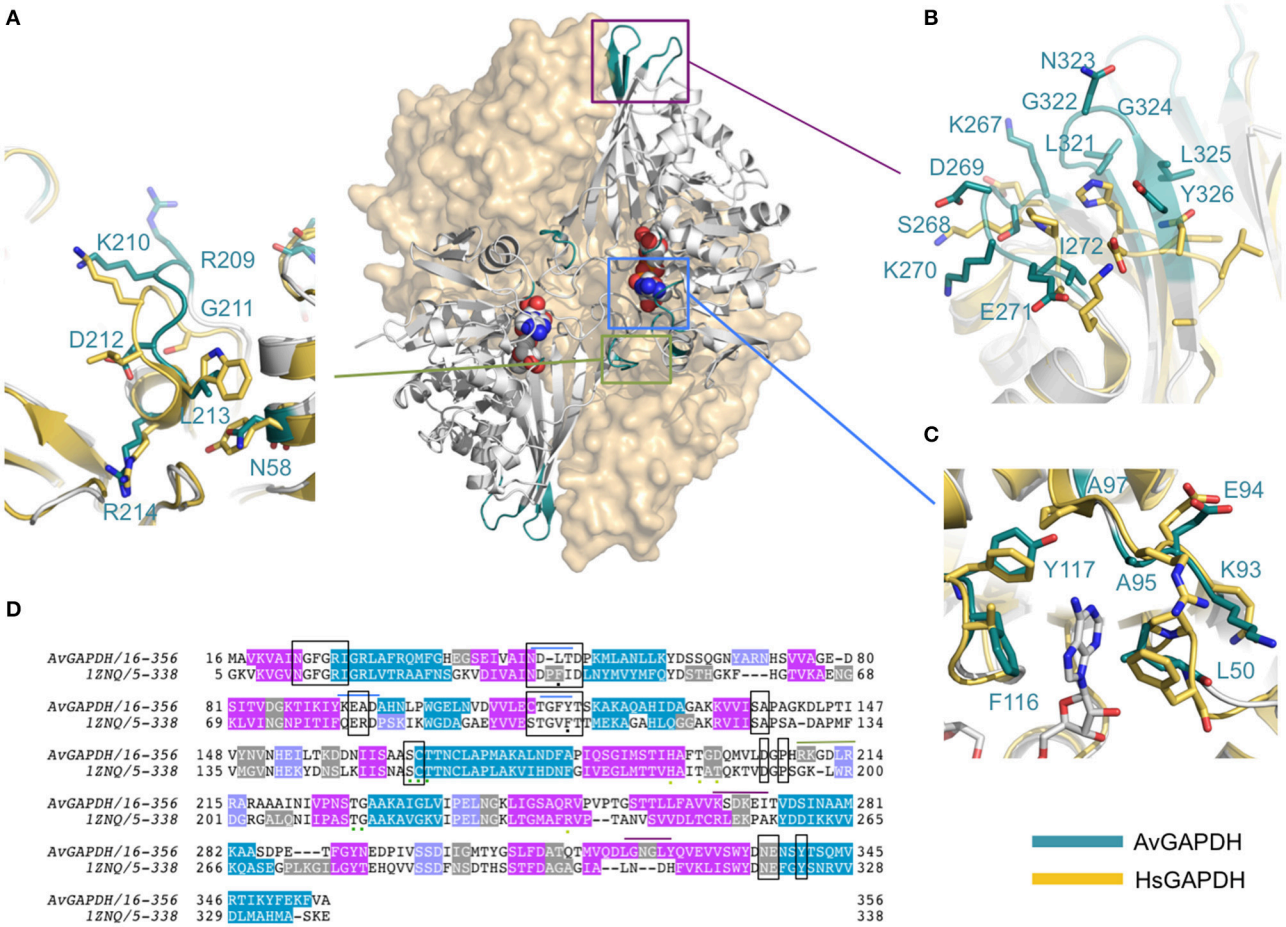
**Structural comparison of ***Av***GAPDH and human liver GAPDH**. Ribbon representations of the *Av*GAPDH and human liver GAPDH (PDB ID 1ZNQ) holoenzymes in gray. The molecular surfaces of two subunits are shown in wheat color. The NAD^+^ cofactor bound to the two subunits represented as ribbons are shown as sphere models in CPK color. Regions where structural differences are more prominent are shown in teal, boxed, and magnified as panels **(A–C). (A)** *S* loop. **(B)** Structurally distinct loop regions on the outer surface of *Av*GAPDH. **(C)** Adenine ring subpocket of the active site. Several pharmacologically relevant compounds have been discovered that target the adenine ring subpocket of GAPDH from trypanosomatids. The superposition, molecular surface, and **(A–C)** were produced with PyMOL (Schrodinger LLC). **(D)** Sequence alignment including the protein sequences of *Av*GAPDH and human liver GAPDH.

An important structural feature of the active site of GAPDH is the *S* loop, a long winding region folded into an irregular shape along the *R* dyad groove spanning residues Ala196-Ile223 (Figures 3A,B) around the *P* dyad (Biesecker et al., 1977; Skarzyński et al., 1987). Residues in the *S* loop contribute to a complex interaction network that includes hydrophobic and ionic contacts around the *R* axis and ionic contacts around the *P* dyad (Skarzyński et al., 1987; Biesecker et al., 1977). Pro207 forms one side of the binding pocket for the adenine-ribose moiety of the NAD^+^ bound to a neighboring subunit related by the *R* axis (Figure 3A). Despite the overall conservation of the peculiar *S* loop sequence in *Av*GAPDH, there are several substitutions plus a Gly211 insertion at Ile203-Arg217 in the middle of the *S* loop (Figure 3B). These modifications cause the protrusion of a turn containing both positively charged side chains (Arg209 and Lys210) toward the crevice where the nicotinamide-ribose moiety of NAD^+^ is located, whereas in most GAPDHs the equivalent turn contains only one conserved Lys (Lys197 in human liver GAPDH; Figure 4A). Electron density for the side chains of Arg209 and Lys210 is weak (Figure 3B), which is attributed to the high thermal parameters of side-chain atoms (backbone atoms have low *B*-factors). In particular, there is no visible electron density for the Cγ-Nε atoms of Arg209 and Cε-Nζ atoms of Lys210 at 1 σ contour level, although the clearly visible electron density for the first side-chain atoms (Cα-Cβ-Cγ) unambiguously (Figure 3B). The Cα atoms of Arg209 and Lys210 are roughly equidistant (14.1−15.6 Å and 12−13.1 Å) from the N7 atom of the adenine moieties in the cofactors bound to the *O* and *R* subunits. The closest distance between either side chain in a fully extended conformation and NAD^+^ is 7.5 Å. In addition, residues Leu204 and Asp205 are conserved and interact with the cofactor through water mediated and main chain interactions (Figure 4A and Supplementary Figure 3). It is remarkable the conservation between the human and the *Av*GAPDH enzymes at and around the entry gateway to the adenine ring of NAD^+^, in particular Val101 (Phe116) and Phe37-Leu50 (Figures 3A, 4C). A Phe in place of Val101 should present steric clashes with Pro82 and Ala97 in *Av*GAPDH, therefore it appears that this change in sequence has a structural role.

#### Structural similarity with SaGAPDH

The closest structural homolog of *Av*GAPDH is *Sa*GAPDH (PDB ID 3K73; Mukherjee et al., 2010) with an r.m.s.d. value of 1.35 Å when the complete quaternary structures are superimposed (0.96 Å on average when each pair of monomers are superimposed), and 63% sequence identity. The structures of *Av*GAPDH and *Sa*GAPDH are therefore considerably conserved, with most differences concentrating in loops and connection regions. For instance, the α1-β_B_ junction is different in the two enzymes, leading to a retraction of the peptide chain that causes a broadening of a solvent-exposed groove over the active site in *Av*GAPDH. In addition, several 3_10_ helices in *Av*GAPDH are absent in *Sa*GAPDH, which are responsible for some local structural deviations; for example, 3_10_A (residues 69−71), which protrudes toward the interface between the two adjacent subunits, has a maximum displacement to the corresponding segment in *Sa*GAPDH of 4.7 Å. On this face of *Av*GAPDH, the structural differences at helix 3_10_A are further amplified by additional local changes at the loop connecting the following strands (β_B_-β_C_). Likewise, the loop spanning Gly141-Asp143 shifts toward the entryway to the active site, contributing to the closing of the NAD^+^-binding pocket, with a maximum distance deviation of 5.5 Å at the center of the loop. Finally, concerted changes affecting the β_L_-α6 loop and the strands β_O_ and β_*P*_ (spanning residues 266−287 and 319−326, respectively) in a fully solvent exposed area lead to severe main-chain displacements as large as 4.29 Å.

#### Kinetic analysis

To compare the catalytic efficiency of *Av*GAPDH with *Sa*GAPDH and other homologous enzymes, we measured its kinetic parameters for the direct reaction by systematically varying NAD^+^, G3P, and P_i_ concentrations. The results are shown in Table 2. For G3P, for which there are more complete kinetic data for *Sa*GAPDH, the *k*_cat_ rate constants of the two enzymes were pretty similar; however, the 10-fold higher *K*_M_ for G3P determined for *Av*GAPDH in comparison with that of *Sa*GAPDH resulted in a correspondingly 10-fold lower *k*_cat_/*K*_M_ ratio. It is interesting than the *K*_M_ for NAD^+^ is smaller than for the co-substrate, G3P, by a factor of 30.

**Table 2:** **Kinetic parameters**.

		***K*_M_**	***V*_M_**	***k*_cat_**	***k*_cat_/*K*_M_**
**GAPDH**	**Substrate**	(mM)	(mM s^−1^ mg^−1^)	(s^−1^)	(mM^−1^ s^−1^)
***A. vaginae***	**G3P**	2.6 ± 0.5	5.7 ± 0.3	57 ± 6	22 ± 5
	**NAD**^+^	0.08 ± 0.03	3.1 ± 0.2	31 ± 3	384 ± 150
	**P_i_**	3.4 ± 0.6	2.9 ± 0.2	29 ± 3	8 ± 2
***S. aureus***^a^	**G3P**	0.32	n.d.	70	218.8
	**NAD**^+^	0.316	n.d.	n.d.	n.d.
	**P_i_**	2.57	n.d.	n.d.	n.d.

a *The kinetic parameters of SaGAPDH were taken from Mukherjee et al. (2010)*.

#### *Av*GAPDH interacts with the human C5a anaphylatoxin

In addition to the essential glycolytic role of *Av*GAPDH, we investigated whether *Av*GAPDH could function as a virulence factor by sequestering the human C5a anaphylatoxin, a complement factor 5 (C5)-derived proteolytic activation fragment which diffuses out of the area wherein C5 is activated to chemoattract neutrophils and macrophages (Marder et al., 1985; Guo and Ward, 2005). This C5a-inhibitory function had been previously observed for *S. pyogenes* (*Sp*) GAPDH (Terao et al., 2006), and it requires the translocation of GAPDH to the cell wall or the extracellular medium, a process that has been described for GAPDH enzymes from a variety of prokaryotic and eukaryotic organisms (Aguilera et al., 2012). Although it is still unknown whether *Av*GAPDH is displayed on *A. vaginae* cells, it is a conceivable scenario that could play an important function in the long-term survival strategy of the pathogen. Since the *Av*GAPDH and *Sp*GAPDH sequences share a 60% sequence identity, we investigated whether both proteins could also share the immunoevasive function described for the *S. pyogenes* enzyme.

**Figure 5:**
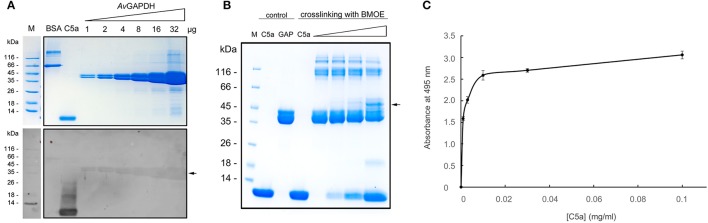
*****Av***GAPDH interacts with human C5a. (A)** Detection of C5a interaction by full-length *Av*GAPDH in ligand blotting experiments. Top, Coomassie brilliant blue (CBB)-stained SDS-PAGE gel where BSA (negative control), biotinylated C5a (positive control, detectable without added biotinylated C5a), and increasing amounts of pure *Av*GAPDH (1−32 μg) were separated. Bottom, ligand blotting was conducted after the electrotransfer of the same SDS-PAGE gel shown on top onto nitrocellulose membrane by incubating the membrane with biotinylated C5a and developing the blot with horse radish peroxidase (HRP)-conjugated anti-biotin antibody and luminol. **(B)** Crosslinking of *Av*GAPDH and C5a with BMOE shown on a CBB-stained SDS-PAGE gel loaded with mock-treated control samples (C5a and *Av*GAPDH, GAP) and BMOE-treated crosslinked samples (increasing concentrations of C5a for a fixed concentration of *Av*GAPDH). The two first lanes with added BMOE represent internal controls for C5a (maximum load) and *Av*GAPDH (no C5a). In **(A)** and **(B)** arrowheads point to the bands where *Av*GAPDH and C5a co-localize. **(C)** C5a bound to *Av*GAPDH immobilized on a polystyrene microtiter plate measured by an ELISA assay. The absorbance at 495 nm is plotted against increasing C5a concentrations. Data represents mean ± *SD* (standard deviation; *N* = 3).

In order to test the possible interaction between *Av*GAPDH and C5a we conducted three sets of experiments. Firstly, we evaluated whether biotinylated C5a could bind to membrane-immobilized *Av*GAPDH in ligand blotting experiments, which have been used before to demonstrate an interaction between *Sp*GAPDH and C5a (Terao et al., 2006). The results show that biotinylated C5a accumulates rather specifically onto *Av*GAPDH bands in a quantitative fashion (Figure 5A), thereby indicating that a complex between membrane-immobilized *Av*GAPDH and biotinylated C5a does indeed form. This is to be expected of an interaction between the fluid-phase C5a and cell wall-associated *Av*GAPDH. In comparison, it is noteworthy the absence of a signal on the negative control (BSA lane) and the strong signal from the positive control (C5a lane, which contains biotinylated C5a).

Secondly, we tested the *Av*GAPDH-C5a interaction in solution using natively folded *Av*GAPDH with the highly specific cross-linking reagent BMOE, which tethers together Cys residues that lie in close proximity (typically, within 8 Å). As shown in Figure 5B, cross-linked *Av*GAPDH-C5a bands developed that prove that a native interaction exists between *Av*GAPDH and C5a that involves surfaces containing a Cys residue in *Av*GAPDH and the only free Cys in C5a (Cys704). Treating C5a or *Av*GAPDH alone with BMOE confirmed that no cross-linking species is generated, thereby ensuring the specificity of the interactions captured by BMOE. However, increasing C5a amounts led to the progressive appearance of a C5a-*Av*GAPDH complex, which runs with an apparent molecular mass *ca*. 10 kDa greater than *Av*GAPDH (Figure 5B, arrow), corresponding to the mass of C5a. As the electrophoretic band containing cross-linked C5a-*Av*GAPDH species becomes more intense, the amount of free *Av*GAPDH becomes lower in proportion, further arguing in favor of a quantitative interaction. In parallel, the amount of free biotinylated C5a unreacted with BMOE is significantly lower than in the corresponding BMOE-treated control lane, thus indicating a proportional reduction of both free species as the amount of cross-linked complex increases. While the involvement of Cys704 of C5a uniquely defines the interaction surface for C5a, there remains an ambiguity in assigning the surface area on *Av*GAPDH engaged for interaction with C5a. While the N-terminal fragment 1−165 of *Sp*GAPDH was sufficient to elicit the interaction with C5a (Terao et al., 2006), an analogous C-terminally truncated version of *Av*GAPDH (residues 1−185) was only able to bind C5a in the same degree than the full-length enzyme in ligand blotting and cross-linking assays, but not in other functional assays (cf. below). This indicates that the complete *Av*GAPDH-C5a interaction surface may engage a more extensive region of *Av*GAPDH than encompassed by the N-terminal deletion construct.

Finally, we also studied the interaction between *Av*GAPDH and C5a using an enzyme-linked immunosorbent assay (ELISA). In this assay, surface-immobilized *Av*GAPDH was capable of binding C5a in a concentration-dependent manner (Figure 5C), whereas uncoated wells or *Av*GAPDH-coated wells that had not been treated with C5a elicited no significant background signal.

### Inhibition of C5a-specific granulocyte chemotaxis by recombinant gapdh variants

**Figure 6:**
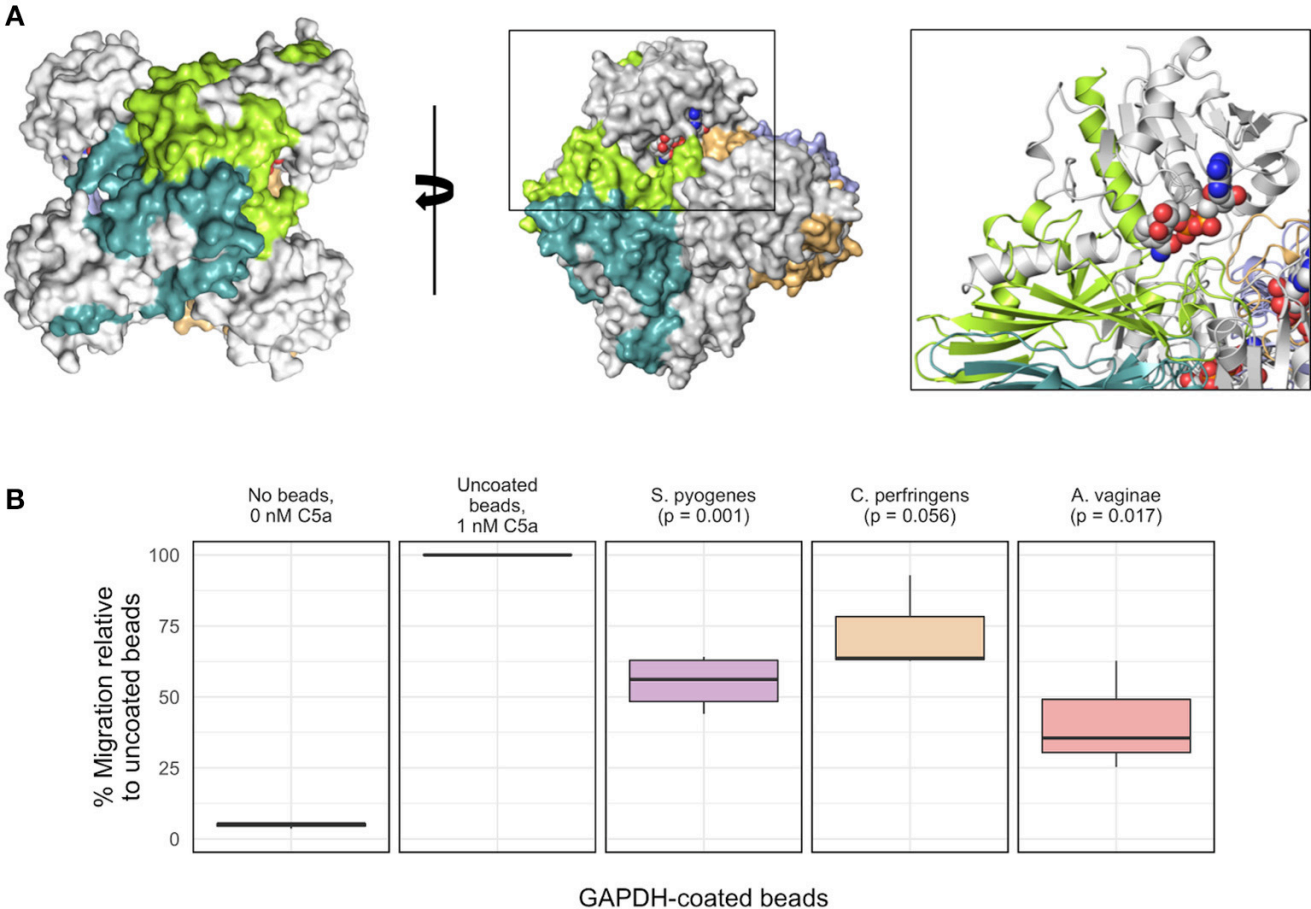
**Inhibition of C5a-specific granulocyte chemotaxis by recombinant ***Av***GAPDH variants. (A)** Molecular surface of the *Av*GAPDH tetramer in two views related by a 90° rotation through a vertical axis. Residues 1−185 of *Av*GAPDH of each protomer, which correspond to the C5a-binding region defined for *S. pyogenes* GAPDH (Terao et al., 2006), are shown in gray, while the remaining C-terminal sequence is shown in chain-specific colors. The black box surrounding one N terminus (NAD^+^ atoms are represented as spheres and colored in CPK) is shown as an enlarged view with *Av*GAPDH structural elements in ribbon representation. **(B)** The boxplot graph shows the results of a transmigration experiment of human granulocytes towards a C5a-containing lower chamber expressed as percent migration (mean ± SEM of 3−4 independent experiments). All results are normalized to the migration of human granulocytes elicited by 0.96 nM C5a incubated with the corresponding amount of uncoated aminoethyl agarose (AM-4) resin beads, taken as 100%; background signal at 0 nM C5a is 4.8%, shown for comparison. The remaining migration experiments were conducted by pre-incubating 0.96 nM C5a with aminoethyl agarose (AM-4) resin beads, either mock (uncoated resin beads) or with covalently immobilized GAPDH molecules. Besides the C5a control, from left to right, the bar graph shows mock AM-4 beads, AM-4 beads with full-length GAPDH from *S. pyogenes, C. perfringens*, and *A. vaginae*. Migration inhibition percentages were normalized to the amount of GAPDH effectively coating the beads. The *p*-values shown above the boxplots for GAPDH-coated beads were calculated with the R software.

To provide further evidence for a potentially immunoevasive function for *Av*GAPH, we next investigated whether *Av*GAPDH was able to inhibit granulocyte (mainly neutrophils) motility, a C5a-specific cellular response that is relevant in the context of bacterial infections. Based on the known interaction surface of *Sp*GAPDH and the binding experiments described for *Av*GAPDH, we would expect that *Av*GAPDH could bind C5a through surface areas located close to the N terminus of *Av*GAPDH (Figure 6A). We tested surface-immobilized *Av*GAPDH by challenging blood-purified human granulocytes in presence of zymosan-activated serum, which contains native human C5a, with increasing concentrations of soluble *Av*GAPDH and two different amounts of *Av*GAPDH-bound resin beads, and measuring the inhibition of neutrophil chemotaxis in a cell migration assay (Figure 6B). The resin-immobilized *Av*GAPDH contained 53 μg/μl full-length *Av*GAPDH (80−85% immobilization yield), therefore providing a dense surface distribution of *Av*GAPDH on the resin beads. As positive controls for both experimental designs, we used immobilized *Sp*GAPDH. Lastly, we also used GAPDH from *Clostridium perfringens* (*Cp*) as a third related protein to assess the capacity of GAPDH enzymes with varying sequences to inhibit C5a-induced cell migration.

The cell migration assays convincingly showed that resin-bound full-length *Av*GAPDH could reduce the amount of C5a-mediated transmigration of granulocytes in our cell-based assay (Figure 6B). The marked reduction in cell migration elicited by preincubating beads coated with *Av*GAPDH (or *Sp*GAPDH) with the activated C5a serum strongly suggested that a direct, physical interaction between *Av*GAPDH and C5a as the likely mechanism for cell motility arrest. The inhibitory effect of *Av*GAPDH on granulocyte migration was significant (*p* = 0.017) and approximately equivalent to that achieved by the same amount of *Sp*GAPDH-immobilized beads (*p* = 0.001), thereby indicating that the neutralization of C5a by *A. vaginae* might be as efficient as during *S. pyogenes* infection. Interestingly, *Cp*GAPDH failed to significantly block granulocyte motility at the concentrations tested (*p* = 0.056), thereby indicating a sequence-dependent effect of GAPDH-mediated inhibition. The varying degrees of cell migration inhibition, in turn, could reflect upon the varying concentrations of C5a in their respective infection sites in humans and their different mechanisms for infection and immunoevasion. Although the amounts of *Av*GAPDH used in these assays are larger than in comparable antagonist-based assays (e.g., Huber-Lang et al., 2002), the increase in avidity caused by the cell surface display of *Av*GAPDH could compensate for the apparent lower binding strength, a binding-potentiating effect that would be even more pronounced during infection, when *A. vaginae* cells typically grow in a highly concentrated biofilm.

The reported interaction between *Av*GAPDH and C5a and the inhibitory effect that *Av*GAPDH has on C5a-induced granulocyte migration may arguably play a role in *A. vaginae* survival during infection by combating pro-inflammatory defense mechanisms mounted by the human immune system. Specifically, colonization and infection of the vaginal tract by *A. vaginae* and other pathogenic bacteria such as *Gardnerella vaginalis* are characterized by triggering an intense inflammatory response with the liberation of interleukins IL-6 and IL-8, which is not elicited by the microbiota of healthy individuals, and that is essential for the pathogenesis (Fichorova et al., 2011; Doerflinger et al., 2014).

### Sequence conservation analysis

**Figure 7:**
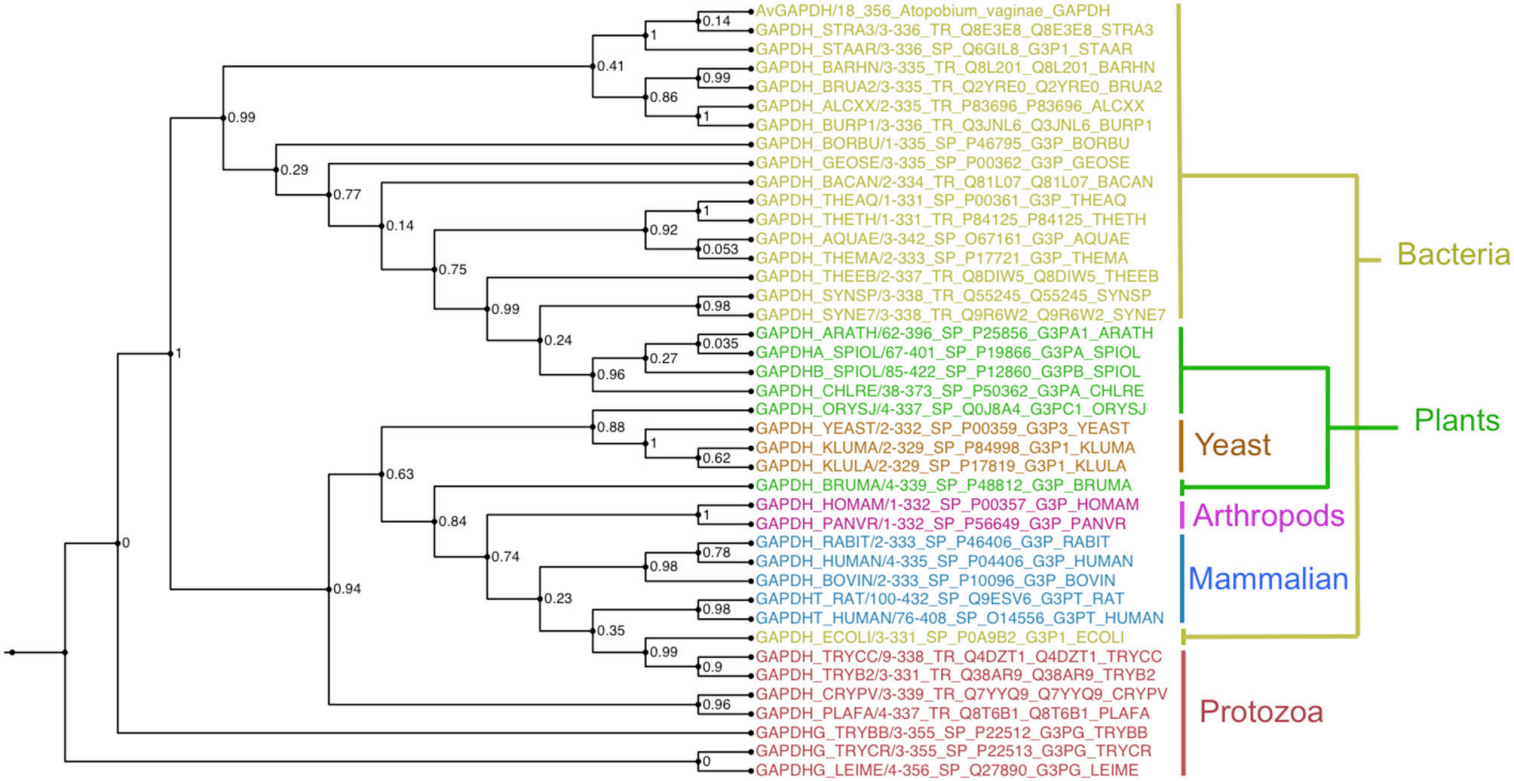
**Evolutionary conservation**. Unrooted phylogenetic tree of selected GAPDH sequences calculated with the maximum-likelihood software PhyML with branch supports shown at every node. Sequences are colored according to taxonomic grouping, with bacteria in yellow, plants and algae in green, yeasts in orange, arthropods in purple, mammals in blue, and protozoa in red. The multiple sequence alignment is provided in the SI. This figure was produced with FigTree (http://tree.bio.ed.ac.uk/software/figtree/).

GAPDH sequences of known structure and with a significant degree of sequence similarity to *Av*GAPDH were found with BLAST (Camacho et al., 2009) using sequences stored in the PDB, aligned with MUSCLE and used to reconstruct a maximum likelihood phylogenetic tree with PhyML (Guindon et al., 2010; Figure 7).

### Implications for *Av*GAPDH-inhibitor design of trypanosomatid GAPDH inhibitors

**Figure 8:**
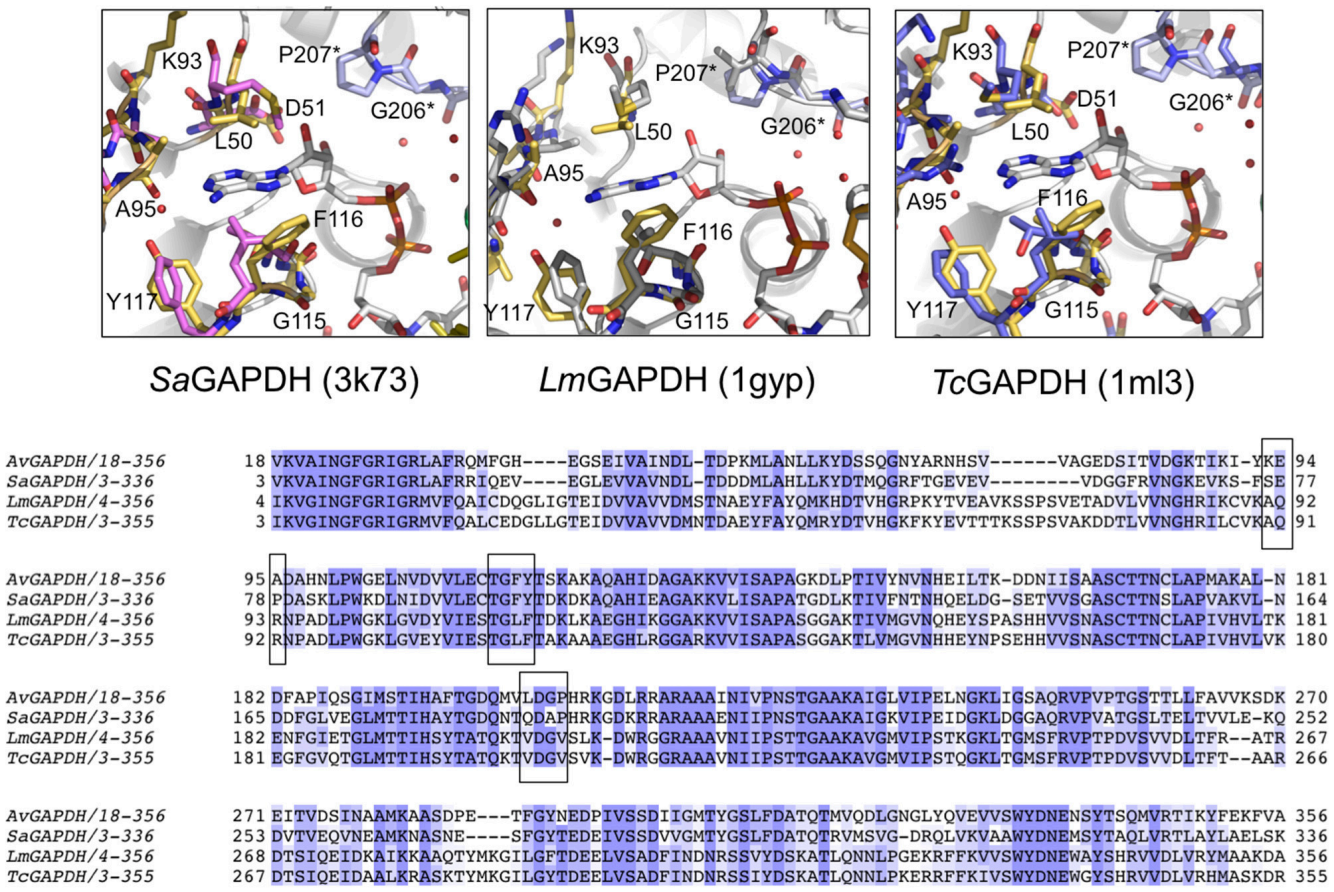
**Comparison of the NAD^**+**^ adenine ring-binding subsite**. Ribbon representation of the NAD^+^ binding pocket in *Av*GAPDH with the cofactor and the residues that lock the adenine ring of the cofactor (in gray) shown in sticks (in gold). Residues from the neighboring chain are labeled with an asterisk and their carbon atoms are shown in violet. **(A)** Comparison with *S. aureus Sa*GAPDH adenosyl-binding pocket (PDB 3K73; pink). **(B)** *L. mexicana Lm*GAPDH (PDB 1GYP; gray). **(C)** *Trypanosoma cruzi Tc*GAPDH (PDB 1ML3; blue). The figure was produced with PyMOL (Schrodinger LLC). **(D)** Multiple sequence alignment of GAPDH from *A. vaginae, S. aureus, L. Mexicana*, and *T. cruzi*. Adenosyl-binding residues are boxed.

In the absence of an efficient treatment for *A. vaginae* infection and the possibility that *Av*GAPDH could participate in the onset and maintenance of the pathogen through both metabolic and immunoevasive strategies, we considered whether available GAPDH inhibitors targeting trypanosomatid GAPDH enzymes, which escape binding to human liver GAPDH, could be used to block *Av*GAPDH. The overall structure of *Av*GAPDH is similar to those of *T. cruzi* (PDB ID 1ML3; Castilho et al., 2003), *T. brucei* (PDB ID 2X0N; Vellieux et al., 1993), and *L. mexicana* (PDB ID 1GYP; Kim et al., 1995), with r.m.s.d. values of 1.35, 1.30, and 1.30 Å over 320 aligned Cα atoms, respectively. Similarly, superposition of *Av*GAPDH with the human and rabbit GAPDH enzymes can be achieved with r.m.s.d. values of 1.26 Å (fully occupied by NAD^+^) and 1.30 Å (devoid of NAD^+^). While most structural differences are located in flexible surface loops, several unique features stand out when the NAD^+^-binding site and, especially, the adenosyl-binding pocket, are compared across GAPDH enzymes from different species (Bressi et al., 2001; Kennedy et al., 2001; Figure 8).

The most notable difference between *Av*GAPDH and the human enzyme concerns the anchoring of the adenine ring into the hydrophobic pocket (Figure 4C). It is precisely the less conserved regions between the GAPDH from pathogens and human GAPDH that are appealing for the design of selective inhibitors, which can impair pathogen viability without compromising the host GAPDH function. Thus, for years, changes in the hydrophobicity of the areas adjacent to the N6 position of the adenine ring and the O2′of the ribose of NAD^+^ molecule have been successfully used as anchor points for selective inhibitors of GAPDH from pathogens such as *Tm*GAPDH, *Tv*GAPDH, and *Tc*GAPDH (Figure 8C). In the crystal structure of *Av*GAPDH, the side chains of Leu50 and Thr114-Gly115-Phe116-Tyr117 surround the NAD^+^ binding pocket around the adenine ring (Figure 8). Indeed, the electron density map contoured at 1 σ level confirms the unusual presence of Tyr117 instead of the highly conserved Phe (Figures 3A, 4C, 8) observed in nearly all sequences, with the exception of rat, human sperm and *Sa*GAPDH (Figure 8A). In all cases, a conserved water mediates the interaction between the OH group of Tyr117 side chain with the N5′ atom in the adenine ring (Figures 8A–C). Furthermore, another feature unique to *Av*GAPDH is the presence of the minimalist loop Ala95-Ala97-Asp96 nearby the position occupied by the adenine ring of NAD^+^ (Figures 3A, 4C, 8A). The intrusion of this loop facilitates the interaction of Ala95 main-chain with the adenine ring. The tandem Phe116-Tyr117 is also missing along several pathogenic bacteria, both Gram-positive (e.g., *Staphylococcus* and *Streptococcus* spp. and *Bacillus anthracis*) and Gram-negative (e.g., *Bartonella, Brucella, Borrelia*, and *Synechococcus* spp.). Also, GAPDH from some thermophilic bacteria (e.g., *Thermus, Aquifex*, and *Thermosynechococcus* spp.) lack an equivalent residue to Tyr117. These differences can be exploited for the design of drugs targeting the N6 position of the adenine ring (Figure 7). On the other hand, the ribose ring of the NAD^+^ cofactor interacts with Pro207, which is conserved in *Hs*GAPDH (Figure 3A). The narrow groove between the Pro207 and Pro36-Phe37 is a distinguishing feature used to search for selective drugs (Figure 3A). A relevant example is 2′-deoxy-2′-(*m*-methoxybenzamido)adenosine (MBDA), a curved molecule that binds selectively to *Tc*GAPDH while it does not bind to *Hs*GAPDH due to steric hindrance (MBDA; 50). For *Av*GAPDH, the equivalent residue is Phe30 and Leu50-Thr51, whose Cα's are 0.5 Å are displaced between them. This will probably make NAD^+^ inhibitors derived with substitutions in O2′ position less effective.

The functional cavities and interfaces of *Av*GAPDH that are most divergent with respect to the human GAPDHs suggest themselves as potential targets for drug discovery. In *Av*GAPDH the hydrophobic residues that surround the adenine ring have a distribution resembling more closely the configuration observed in the glycosomal GAPDHs than the human liver enzyme. Specifically, Leu50 occupies in *Av*GAPDH the position of Pro36-Phe37 in the human liver GAPDH and Thr114, Phe116, and Tyr117 shield the opposite side of the adenine pocket (Figure 4C and Supplementary Figure 5). Based on this observation, other strategies than those used for *Tv*GAPDH, *Lm*GAPDH, and *Tc*GAPDH have to be adopted to inhibit *Av*GAPDH, and we propose that nucleoside analogs such as the adenosine derivatives like MBDA (MBDA; 50) represent an interesting starting point for future developments.

## Discussion

### Crystal structure of *Av*GAPDH

The crystal structure of *Av*GAPDH reported herein at 2.19 Å has been refined to convergence with *R*_work_/*R*_free_ = 0.177/0.226 and the final model exhibits excellent stereochemistry (Table 1). Although the overall fold of *Av*GAPDH is highly conserved when compared with reported GAPDH structures (Figure 1), there are distinctive structural features in *Av*GAPDH with important consequences for potential protein binding partners as well as for the discovery of selective inhibitors (Figures 4, 8). The availability of the first high-quality, high-resolution crystal structure of a complement immunoevasive GAPDH can generate insights into the mechanistic basis for complement interference as well as provide a template for inhibitor search.

As previously reported, streptococcal GAPDH can function as an immunoevasion factor by sequestering *in-situ* produced C5a on the pathogen's surface during the lytic or terminal phase of complement activation (Terao et al., 2006). Terao et al. concluded that the N-terminal region of *Sp*GAPDH (residues 1−165) was the likely C5a-recognition motif (Terao et al., 2006). The homologous region in *Av*GAPDH spans residues 1−185 and is reasonably well conserved, with 56.4% identity and 66.4% similarity (compare with the full-length sequence alignment, with 64.9% identity and 70.7% similarity). Since the structure of the N-terminal region of GAPDH enzymes is partly defined by the NAD^+^-binding pocket, these loops share an overall structural organization and they differ mostly by the array of residues that become solvent exposed. In particular, the structure of residues 1−185 in *Av*GAPDH and the equivalent segment of the closely related *Sa*GAPDH (Mukherjee et al., 2010) trace roughly equivalent spatial trajectories over and above the cofactor-binding site. This is the proposed C5a-docking surface, which provides a relatively flat and flexible platform that could accommodate the binding of the small C5a molecule.

This structure and sequence-backed findings suggest that GAPDH enzymes from pathogenic bacteria located over widely diverse evolutionary distances could however be equipped with conserved C5a-binding surfaces. Since *S. pyogenes, A. vaginae*, and *S. typhimurium* are all important human pathogens, the immunoevasive capacity detected for bacterial GAPDH might conceivably have been selected for in the context of infection. If this hypothesis holds true, it would imply that the pattern of C5a sequestration by bacterial GAPDH should be extended to a large diversity of Gram-positive and Gram-negative bacterial pathogens. A second implication would consist in the opening of a window of opportunity for the treatment of antibiotic-resistant bacterial infections with complement stimulatory therapies, e.g., by releasing C5a blockade.

### *Av*GAPDH as a complement immunoevasive factor through C5a

The relatively high sequence identity and similarity shared between *Sp*GAPDH and *Av*GAPDH suggested that the latter could also elicit C5a sequestration. The experimental evidence presented hereby corroborates a likely role for *Av*GAPDH as an immunoevasive factor. Of the experiments performed, ligand blotting using biotinylated-C5a (Figure 5A) had been previously used with *Sp*GAPDH to establish its interaction with C5a (Terao et al., 2006). In addition, we have carried out highly specific cross-linking experiments with BMOE to prove a physical interaction between *Av*GAPDH and C5a (Figure 5B). BMOE crosslinks protein thiol groups <8-Å apart, thereby completely avoiding spurious covalent complexes. Additional ELISA experiments corroborated the significance of the *Av*GAPDH-C5a interaction originally identified by the ligand blotting and cross-linking experiments. The previous three *in vitro* approaches converged on a model whereby *Av*GAPDH and C5a can form a complex. Unfortunately, we could not isolate a stable complex by chromatography to subject it to structural analysis. Given the evidence at hand and that from previous studies (Terao et al., 2006), we attribute this behavior to the complex environment wherein this interaction will occur in vivo, which might potentiate the physical association and/or provide a more favorable context for the interaction (e.g., on the cell-wall surface). Future research should tackle the issue of context and presentation for the host-pathogen interaction described in this work.

To seek independent verification for the *Av*GAPDH-C5a interaction, we tested the ability of soluble and surface-immobilized *Av*GAPDH, *Cp*GAPDH, and *Sp*GAPDH to inhibit neutrophil chemoattraction, which was triggered with native C5a generated *in situ* by incubating C5-containing serum with zymosan (Figure 6B). Since at least *Av*GAPDH and *Sp*GAPDH are C5a interactors, the most likely mechanism for neutrophil migration blockade is physical sequestration of C5a by its binding partners. The results convincingly show that *Av*GAPDH and, to a similar extent, *Sp*GAPDH, are capable of reversing the C5a-induced chemoattraction of neutrophils in a cell migration assay. *Cp*GAPDH, in contrast, was less effective than either *Av*GAPDH or *Sp*GAPDH in sequestering C5a. The observed inhibition was markedly stronger when surface-immobilized *Av*GAPDH was used as compared with the effect of soluble proteins, thereby indicating that the precise structural-chemical context where the GAPDH-C5a interaction can occur is bound to play a significant biological role. The fact that undecorated agarose beads were incapable of blocking neutrophil migration rules out an inhibitory role for the carrier material.

When combined, the ligand blotting, cross-linking, ELISA interaction assays, and cell migration inhibition assays together offer a coherent view of *Av*GAPDH (and *Sp*GAPDH) as a virulence factor. In one of its manifold moonlighting functions, secreted and/or surface-immobilized *Av*GAPDH bears the potential to titrate out serum C5a from the infected areas where the anaphylatoxin is generated. As such, this complement down-regulatory mechanism could be at play in those cases where BV has been associated with a strong pro-inflammatory response as a result of the secretion of IL-6 and IL-8 (Fichorova et al., 2011; Doerflinger et al., 2014).

### GAPDH as a chemotherapeutic target for *A. vaginae*

The two most salient differences between GAPDH from *A. vaginae* and the human liver enzyme are localized in the adenosine binding subpocket of NAD^+^ and around the *S* loop that faces it (Figure 4). These and other differences have been exploited for the successful design of anti-GAPDH compounds against trypanosomatids that escape trapping in the liver. In a similar manner, we reason that the observed differences could be exploited to discover compounds (or reuse anti-trypanosomatid GAPDH drugs) against *Av*GAPDH. This is a pressing matter owing to the inefficiency of current therapeutic options for the treatment of BV caused by the swift appearance of antibiotic resistant bacterial strains. When the *Av*GAPDH structure is compared with those of trypanosomatids, the pattern of conserved and diverging structural elements presents differences with those described for the comparison with the human liver enzyme (Figure 8). Interestingly, naïve chemical modeling of known anti-trypanosomatid GAPDH inhibitors into the *Av*GAPDH active site suggests that these well-studied families of compounds could be effective as inhibitors and, therefore, represent new therapeutic opportunities for BV.

The significance of the high-resolution *Av*GAPDH structure for drug discovery initiatives targeting human BV might be large if *A. vaginae* infections rely greatly on a functional glycolytic pathway. In principle, an effective anti-GAPDH agent that binds into the active site could as well disrupt the long loop that covers the NAD^+^ cofactor and is involved in C5a binding, therefore suggesting the interesting possibility that such an inhibitor could both down-regulate the glycolytic flux and also perturb C5a recognition by *Av*GAPDH.

Future investigations will address whether compounds related to the available anti-trypanosomatid GAPDH drugs can indeed effectively inhibit *Av*GAPDH.

### Is complement immunoevasion a widely shared moonlighting property of GAPDH?

There is accumulating persuasive evidence that metabolic enzymes, especially abundant enzymes such as the glycolytic enzymes GAPDH and enolase, are able to perform important functions unrelated to their cognate catalytic activity (Terao et al., 2006; Agarwal et al., 2012; Terrasse et al., 2012; Wang and Jeffery, 2016). These so-called moonlighting functions of predominantly cytosolic enzymes are typically deployed at specific subcellular compartments or locations (e.g., cell nucleus, mitochondria, plasma membrane) or in extracellular contexts. The underlying causes that would explain how glycolytic enzymes came to develop these functions are not yet fully appreciated, although it is now clear that these additional functions have been selected for during evolution and are not merely a side effect of mislocalization or chance occurrence. Most of the hypotheses that have been advanced include the high concentration of these enzymes as a driver factor for their selection to fulfill accessory functions in particular contexts, under the rationale that reassigning existing genes and proteins for new functions is economical and efficient. This strategy appears particularly compelling for genome-encoded virulence factors, where two selective pressures (growth inside the host and immunoevasion) become coupled through a very abundant, multifunctional enzyme like GAPDH.

**Figure 9:**
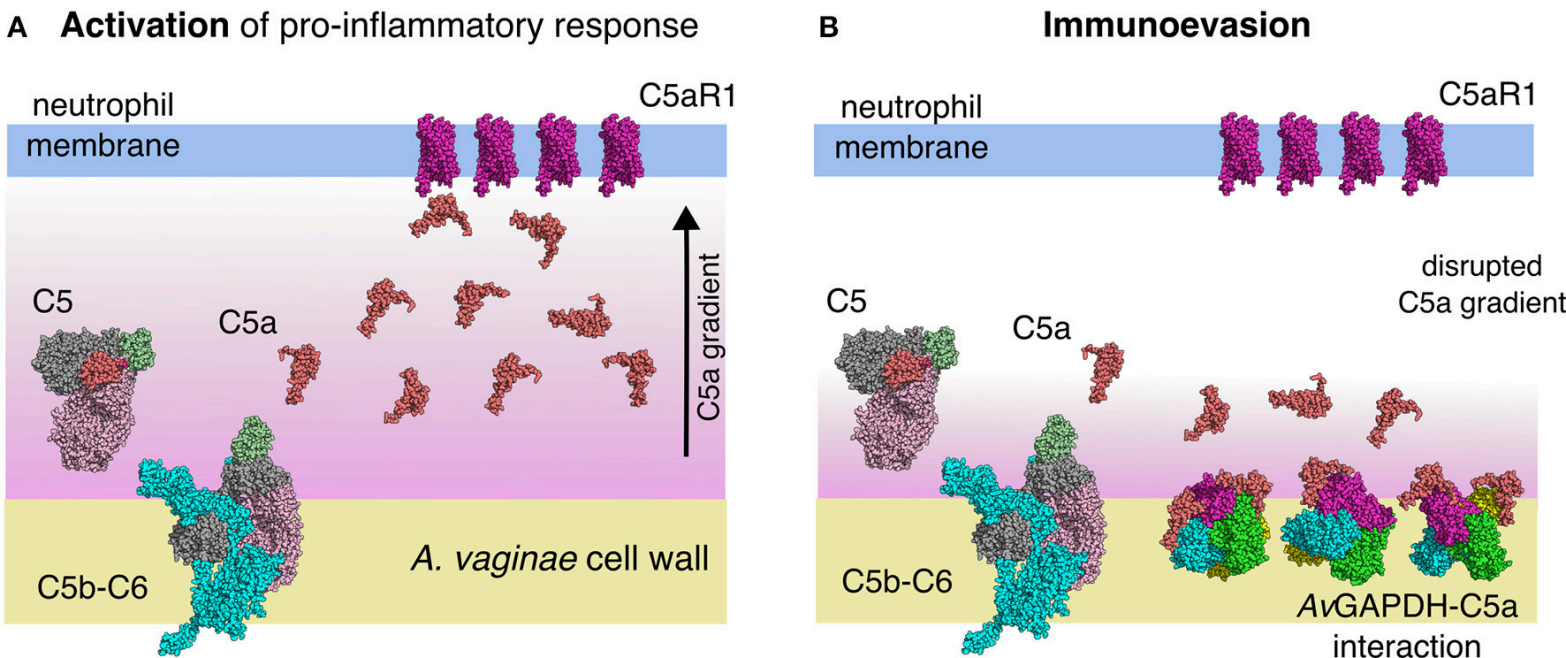
**Model for the immunoevasive function of ***A. vaginae*** GAPDH through C5a sequestration. (A)** A C5a gradient generated by the activation of the lytic phase of complement's alternative pathway leads to the recruitment of neutrophils to the site of infection through the activation of pro-inflammatory signaling cascades via C5a receptor (C5aR1) stimulation. **(B)** Cell-wall associated *Av*GAPDH traps in-situ generated C5a, thereby disrupting the chemotactic gradient and effectively “blinding” neutrophils about the presence of the bacterial pathogen. Macromolecular structures are shown to scale and represented as sphere models. C5 (PDB ID 3CU7) (70) is colored according to its domain structure; C345c is shown in pale green, the C5a anaphylatoxin in red, the β-chain in light pink, and the α-chain in gray. In C5b-C6 (PDB ID 4A5W) (71), C5b is colored as C5 (except for the anaphylatoxin domain, which is missing in C5b) and C6 is colored in cyan. C5a (PDB ID 3HQA) (72) is shown in the same color as the anaphylatoxin domain of C5. The transmembrane region of C5aR1 was modeled the crystal structure of ligand-free rhodopsin (PDB ID 3CAP) (73) without loops and colored in violet.

The involvement of the extracellular GAPDH of *S. pyogenes* in C5a sequestration (Terao et al., 2006) and of the *S. pneumoniae* enzyme in C1q binding (Terrasse et al., 2012) provide reference cases for the existence of complement immunoevasion mechanisms in streptococcal infections that depend on the moonlighting action of GAPDH. The extension of these mechanisms to GAPDH from other Gram-positive pathogenic bacteria that share significant sequence similarity with *S. pyogenes* GAPDH is relevant and intriguing. Firstly, because GAPDH is an essential gene present in all bacteria and could therefore be seen as a universal virulence factor tasked with down-regulating complement-mediated recruitment of neutrophils to sites of infection. Secondly, because sequence similarity across bacterial GAPDH genes cuts across phylogenetic groups and reveals broad patterns of horizontal transfer events, thereby indicating the potential for widespread dispersion of immunoevasive GAPDH sequences. One such example is the high sequence similarity between *A. vaginae* (a Gram-positive bacterium) and *S. aureus* (a Gram-negative proteobacterium), which would suggest a potential for C5a sequestration for *S. aureus* GAPDH. If this were the case, interference with the normal function of the complement anaphylatoxin-mediated signaling could provide a general mechanism for down-modulating the innate immune response. In the context of the infection biology of *A. vaginae*, deploying molecules capable of depressing the spread of anaphylatoxins away from the site of infection would be advantageous for the bacterium to proliferate (Figure 9). In addition, the depletion of C5a would obviously help the proliferation of the complex community of bacteria that cooperate with *A. vaginae* in establishing BV, including *G. vaginalis* and other associated bacteria.

Might then reverting this C5a sequestration mechanism offer therapeutic opportunities against bacterial infections? Bacterial pathogens wreak havoc by the action of specialized virulence factors and by aggressive proliferative behaviors, none of which relies solely on immunoevasive mechanisms. Down-regulating pro-inflammatory responses mediated by C5a can help the pathogen expand at the onset of an infection, whereby escaping the watch of innate immunity may be crucial for its initial survival. Combination therapies attacking other aspects of bacterial pathogenesis while simultaneously freeing the complement system (e.g., C5a and C1q) from interfering influences from bacterial molecules such as GAPDH could result in a more effective strategy, whereby the chance for decimated bacterial populations to endure through the antibiotic treatment and thrive back again as soon as antibiotic treatment stops would be minimized. As further knowledge accumulates as to the potentially beneficial effects of modulating complement protein factors during infections, these strategies should be seriously considered, as their applicability would span a very wide range of bacterial pathogens.

## Accession number

Atomic coordinates and diffraction data of *A. vaginae Av*GAPDH have been deposited at the Protein Data Bank (PDB) with accession code 5LD5.

## Author contributions

CV, JR, SA, and SR conceived the experimental study, designed the experiments, analyzed the data and wrote the manuscript. JQ, FF, CM, and CV expressed and purified *Av*GAPDH and C5a. JQ and FF crystallized *Av*GAPDH. FF collected the X-ray data set from *Av*GAPDH crystals. DF and JJ contributed to the X-ray diffraction data collection. SG measured *Av*GAPDH kinetics and prepared bead-immobilized GAPDH. AM, SR, CV, and JR conceived, designed and conducted the granulocyte migration experiments and analyzed the data. SA provided materials and helped design the experiments and analyze the data. All authors contributed critically to the manuscript. CV wrote the manuscript.

## Funding

The research leading to these results has received funding from the Spanish *Instituto de Salud Carlos III* (PI12/01667 to CV), the Spanish *Ministerio de Economía y Competitividad* (CTQ2015-66206-C2-2-R and SAF2015-72961-EXP to CV, SAF2015-66287-R (MINECO/FEDER) to SR and SAF2014-54708-R to JR), CSIC (201620E064), the Regional Government of Madrid (S2010/BD-2316 to JR, CV, and SR), and the European Commission (Framework Programme 7 (FP7)) projects ComplexINC (Contract No. 279039 to CV) and EURenOmics (Contract No. 305608 to SR). AVM was supported by the *Comunidad de Madrid* (S2010/BMD-2316/2326) and the *Universidad Complutense de Madrid* (CT46/15). The funders had no role in study design, data collection and analysis, decision to publish, or preparation of the manuscript.

### Conflict of interest statement

The authors declare that the research was conducted in the absence of any commercial or financial relationships that could be construed as a potential conflict of interest.
