# Supplementary material for: Crystal Structure of Glyceraldehyde-3-Phosphate Dehydrogenase from the Gram-Positive Bacterial Pathogen A. vaginae, an Immunoevasive Factor that Interacts with the Human C5a Anaphylatoxin
Source: Front Microbiol. 2017 Apr 10;8:541. doi: 10.3389/fmicb.2017.00541 (PMC5385343; doi:10.3389/fmicb.2017.00541)
Supplement: Supplementary file 1 [file Image1.PDF]

Supplementary Material

Crystal Structure of Glyceraldehyde-3-phosphate Dehydrogenase from the Gram-positive Bacterial Pathogen *A. vaginae*, an Immunoevasive Factor that Interacts with the Human C5a Anaphylatoxin

Javier Querol-García, Francisco J. Fernández, Ana V. Marín, Sara Gómez, Daniel Fullà, Cecilia Melchor-Tafur, Virginia Franco-Hidalgo, Sebastián Albertí, Jordi Juanhuix, Santiago Rodríguez de Córdoba, José R. Regueiro, and M. Cristina Vega*

*** Correspondence:** Corresponding Author: cvega@cib.csic.es, cristina.vega@strubicib.org

1 Supplementary Figures

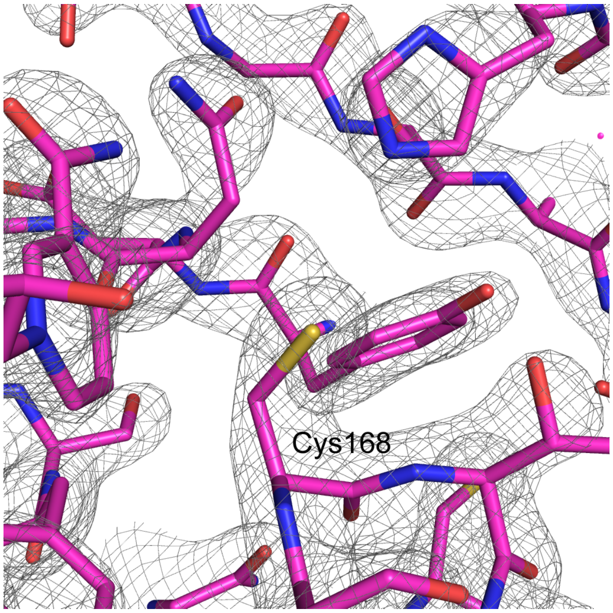

Supplementary Figure 1. Fully reduced state of the catalytic Cys168 of *Av*GAPDH. Unbiased omit map calculated with molecular replacement phases (prior to building the cofactor and solvent structures and prior to refinement) contoured at 1.5 σ illustrating the complete absence of any excess electron density around the catalytic Cys168 of all subunits (here the Cys168 residue of chain Q is shown), therefore attesting to the fully reduced state of the highly reactive side-chain thiol moiety of Cys168 (thiol in yellow, close to the center of the image). Otherwise, the presence of a spontaneous oxidation product such as the sulfonic acid derivative or an *S*-oxyl cysteine would have yielded prominent features in the electron density map beyond the Sγ atom of Cys168 corresponding to additional oxygen atoms, which are clearly lacking.

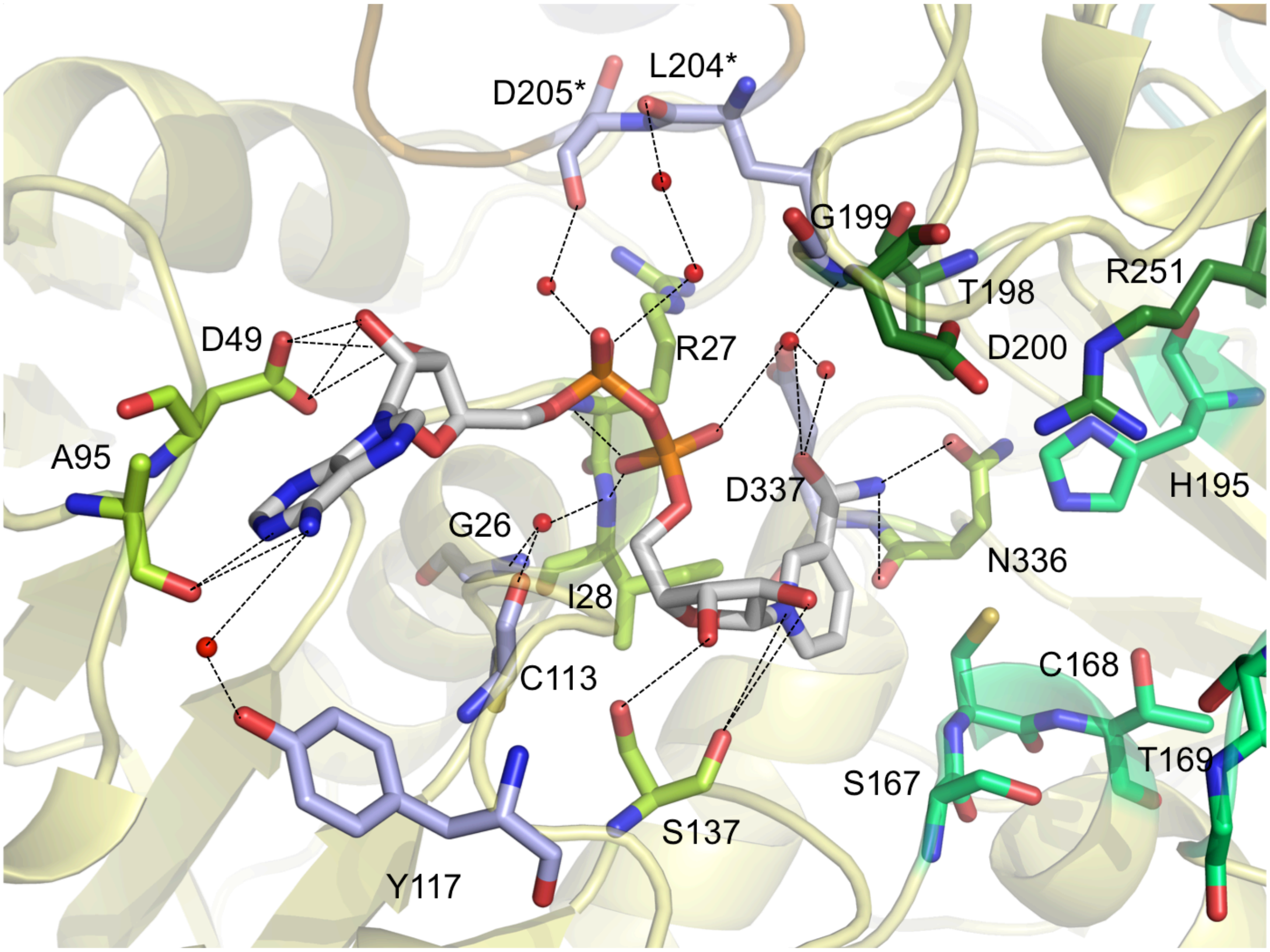

Supplementary Figure 2. Water-mediated interactions of *Av*GAPDH with the NAD$^+$ cofactor. Residues shown in light blue color interact with the NAD$^+$ cofactor via water molecules. The P_s site residues are in dark green and those of the "new P_i" site are in light green.

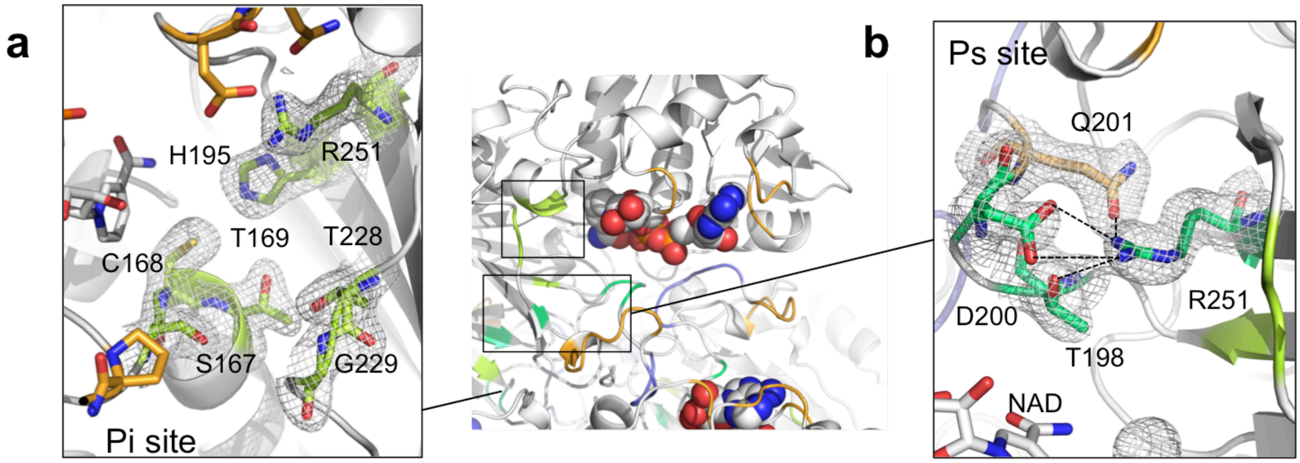

Supplementary Figure 3. Phosphate sites in *Av*GAPDH. The central panel shows a ribbon representation of *Av*GAPDH down one of the subunit interfaces. The NAD^+ cofactor is shown in a sphere representation and CPK colors. Different functionally relevant areas are color coded as follows. Loops harboring cofactor-binding residues and *S* loop residues are shown in wheat (except for such residues in the *S* loop, which are violet). Residues that constitute the P_s site are in dark green. Residues in the P_i site are in light green. (*a*) P_i site. (*b*) P_s site. NAD^+ in the (*a*) and (*b*) insets is represented as sticks with the carbon atoms in gray.

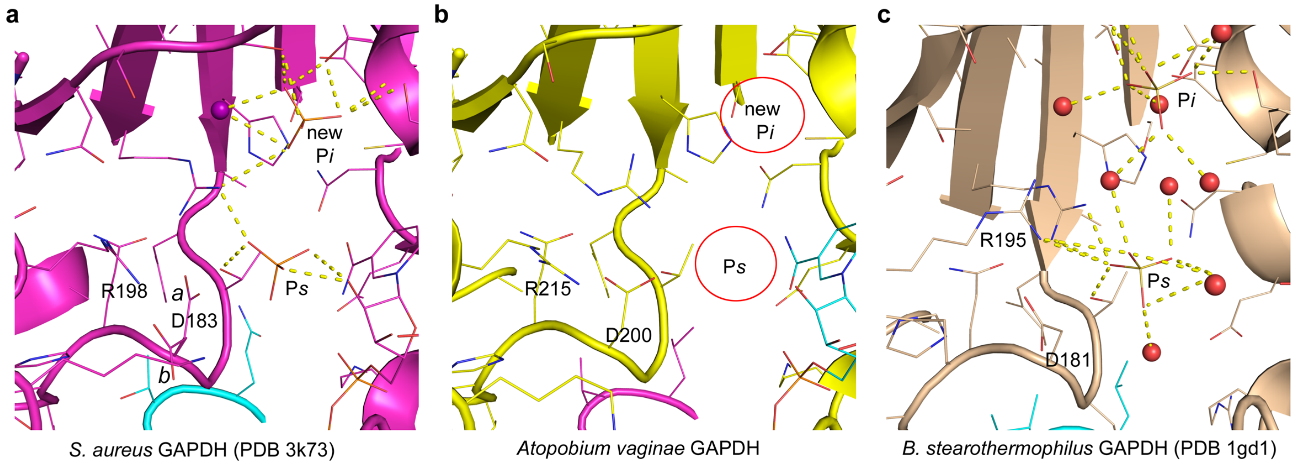

Supplementary Figure 4. The P_s and P_i sites in *Av*GAPDH in comparison with *S. aureus* and *B. stearothermophilus* GAPDH. Ribbon representation of the active site architecture of GAPDH from *S. aureus* (*a*), *A. vaginae* (*b*), and *B. stearothermophilus* (*c*), with relevant amino acid residues depicted as lines (chains and atom types are color coded). The interactions between the bound phosphate ions in (*a*) and (*c*) to protein residues and to water molecules (spheres) are shown as dashed lines. In (*b*), the proposed binding pockets for phosphate in *A. vaginae* GAPDH (PDB ID 5LD5) are indicated by red ellipses. The significant sequence and structural similarity shown by the superposition of the crystal structure of the *Av*GAPDH holoenzyme with that of *Sa*GAPDH (PDB 3k73, the holoenzyme co-crystallized with phosphate ions) indicates that their P_i sites should indeed be very similar, strongly suggesting that a "new P_i" site rather than a "classical P_i" would be used by *Av*GAPDH during catalysis. The side-chain rotamers of Asp200 and Arg215 in *Av*GAPDH are identical to those in *Sa*GAPDH (panel *a*; here there are two alternative conformations for Asp183 are labeled "*a*" and "*b*", one identical to *Av*GAPDH and one to *Bs*GAPDH), while in (*c*) the same rotamers are flipped over in *Bs*GAPDH (PDB 1gd1). In *Sa*GAPDH, the occupancy (1.0) and overall *B*-factor of the P_s phosphate (≈ 80 $Å^2$) suggests that the occupancy of this phosphate ion should have been coupled to that of one of the Asp183 conformations seen in the crystal (occupancy 0.5, overall *B*-factor 47 $Å^2$). Indeed, the atoms immediately surrounding the P_s phosphate including protein, NAD^+, and solvent ions all have *B*-factors between 35-54 $Å^2$, further supporting the notion that the inward rotamer conformation of Asp200 (in *Av*GAPDH nomenclature) would correspond to an empty P_s site, whereas the outward facing rotamer would be consistent with an occupied P_s site.

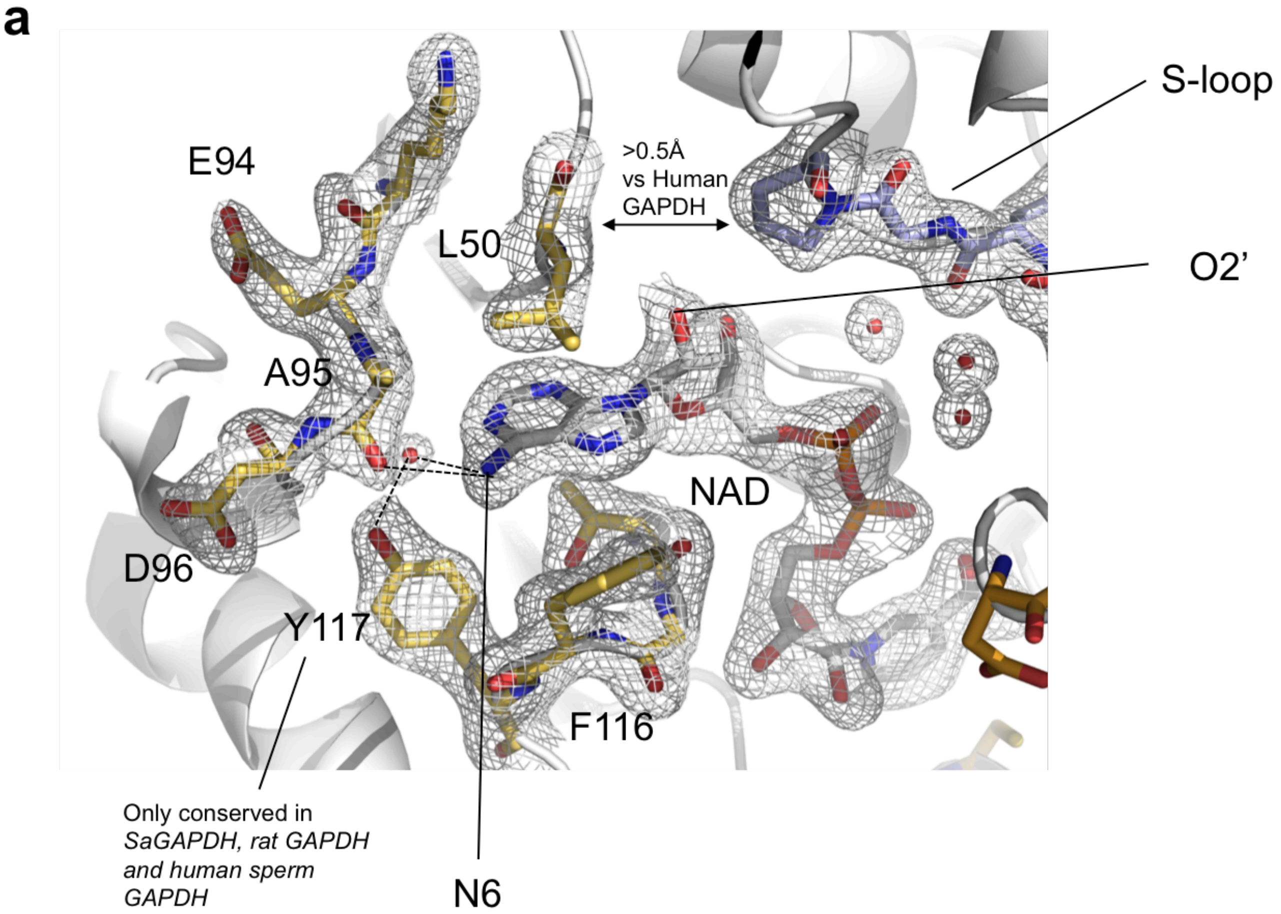

Supplementary Figure 5. Adenosyl-binding subpockets in *Av*GAPDH. The structural elements making up the *Av*GAPDH cofactor pocket are represented as cartoons in gray. NAD^+ is shown as sticks with carbon atoms in gray. Important residues found in the cofactor-binding pocket are represented as sticks with carbon atoms shown in gold (adenosyl-contacting residues, both directly or indirectly via water-mediated hydrogen bonds) or in violet (residues along the *S* loop). The σA-weighted 2*m*Fo−*D*Fc electron density map overlays all key residues and the cofactor and is contoured at 1 σ level. The two subpockets that have been used for drug discovery face either the N^6 atom of the adenylyl ring or the crevice facing the O2′ atom of the adenosine ribose.

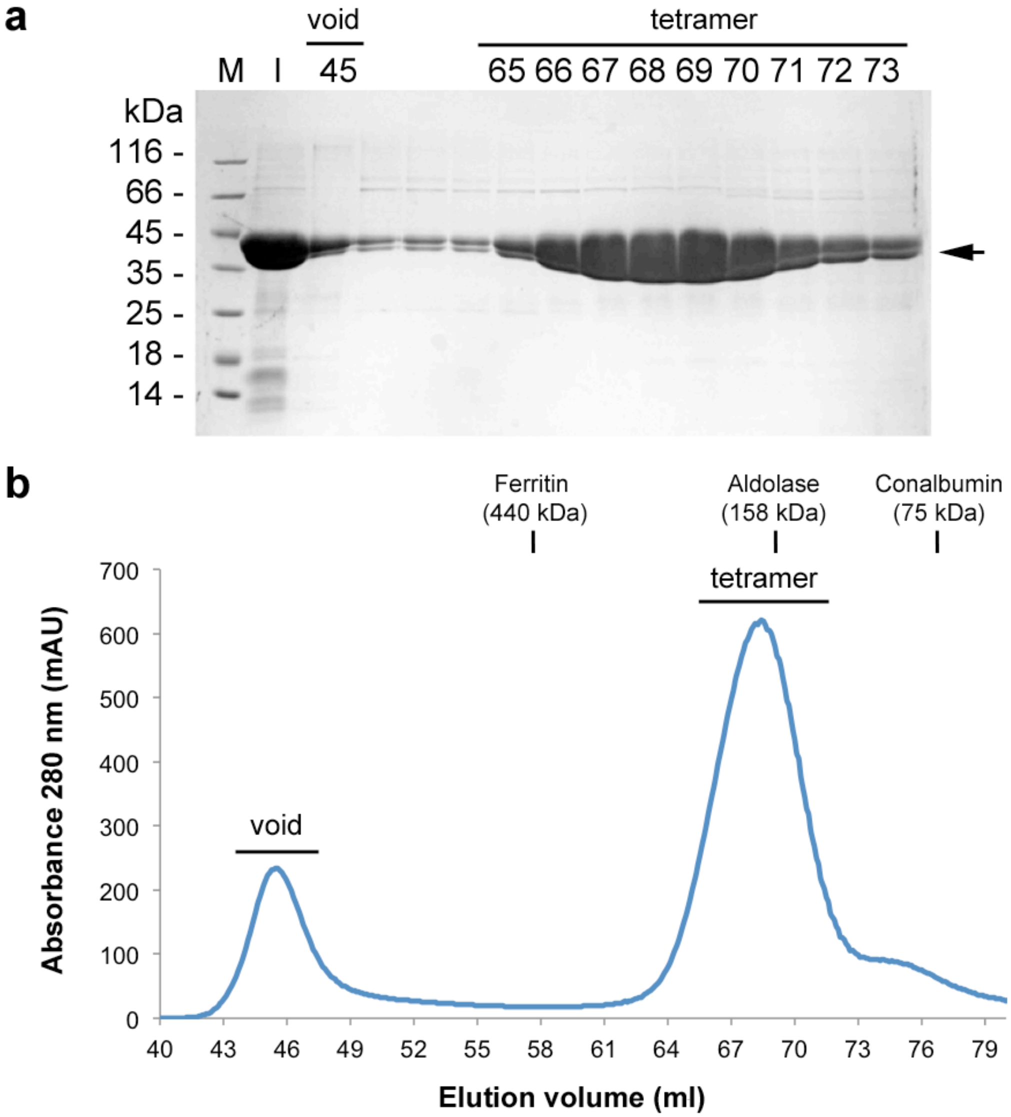

Supplementary Figure 6. Quality of the recombinant *Av*GAPDH protein sample. (*a*) Coomassie brilliant blue (CBB)-stained SDS-PAGE electrophoresis of nickel affinity-purified *Av*GAPDH (>95% pure) further purified by size-exclusion chromatography over a Superdex 200 HR 16/60 column (GE Healthcare). Molecular size ladder is shown to the left, and elution fractions on top of the gel. (*b*) Size-exclusion chromatography profile confirmed that the quaternary structure of *Av*GAPDH corresponds to a constitutive homotetramer. The molecular size was estimated from a calibration curve constructed using high and low molecular weight calibration kits (GE Healthcare); relevant molecular size standards represented over the chromatogram.
